# Supplementary material for: A single transcription factor facilitates an insect host combating Bacillus thuringiensis infection while maintaining fitness
Source: Nat Commun. 2022 Oct 12;13:6024. doi: 10.1038/s41467-022-33706-x (PMC9555685; doi:10.1038/s41467-022-33706-x)
Supplement: Supplementary file 1 — Supplementary Information [file 41467_2022_33706_MOESM1_ESM.pdf]

Supplementary Information

A single transcription factor facilitates an insect host combating *Bacillus thuringiensis* infection while maintaining fitness

Guo et al.

This PDF file includes:

Supplementary Figures 1 to 10

Supplementary Tables 1 to 6

Supplementary Note 1

Other supplementary materials for this manuscript include the following:

Supplementary Data 1

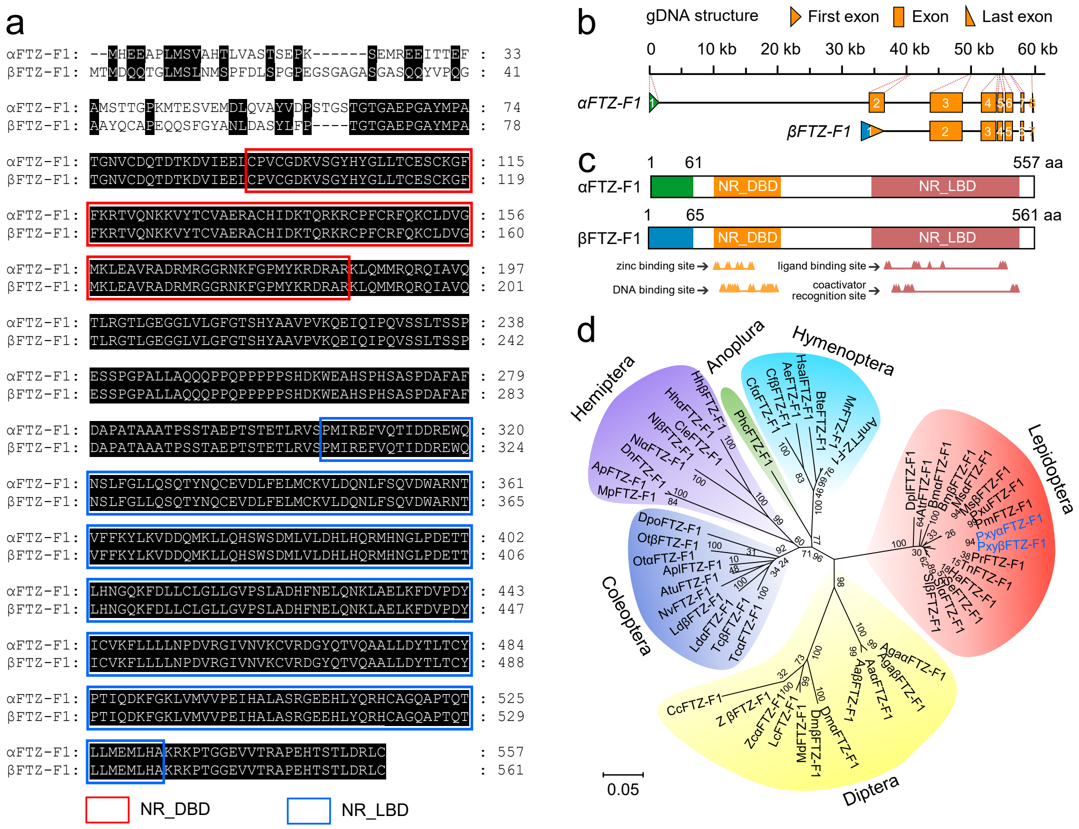

Supplementary Figure 1. Analysis of the structure of the FTZ-F1 in *P. xylostella*. **a** Amino acid sequence alignment of two isoforms of the FTZ-F1 protein in *P. xylostella*. αFTZ-F1 and βFTZ-F1 harbor unique N-terminal sequences that are fused to a common nuclear receptor (NR) DNA-binding domain (DBD) (indicated by red box) and ligand-binding domain (LBD) (indicated by blue box). **b** Genomic structure of the *FTZ-F1* gene. The *FTZ-F1* gene encodes two isoforms: *αFTZ-F1* and *βFTZ-F1*. Colored boxes denote the exons. **c** Schematic diagram of the FTZ-F1 protein structures. αFTZ-F1 and βFTZ-F1 contain two relative conserved domains: the same DBD for recognition and binding of DNA motif and the same LBD for protein dimerization. **d** Phylogenetic analysis of FTZ-F1 in different insects. The full-length cDNA sequences of both αFTZ-F1 and βFTZ-F1 proteins from our work are highlighted in blue. Full-length amino acid sequences of the FTZ-F1 in other insects were retrieved from the GenBank database.

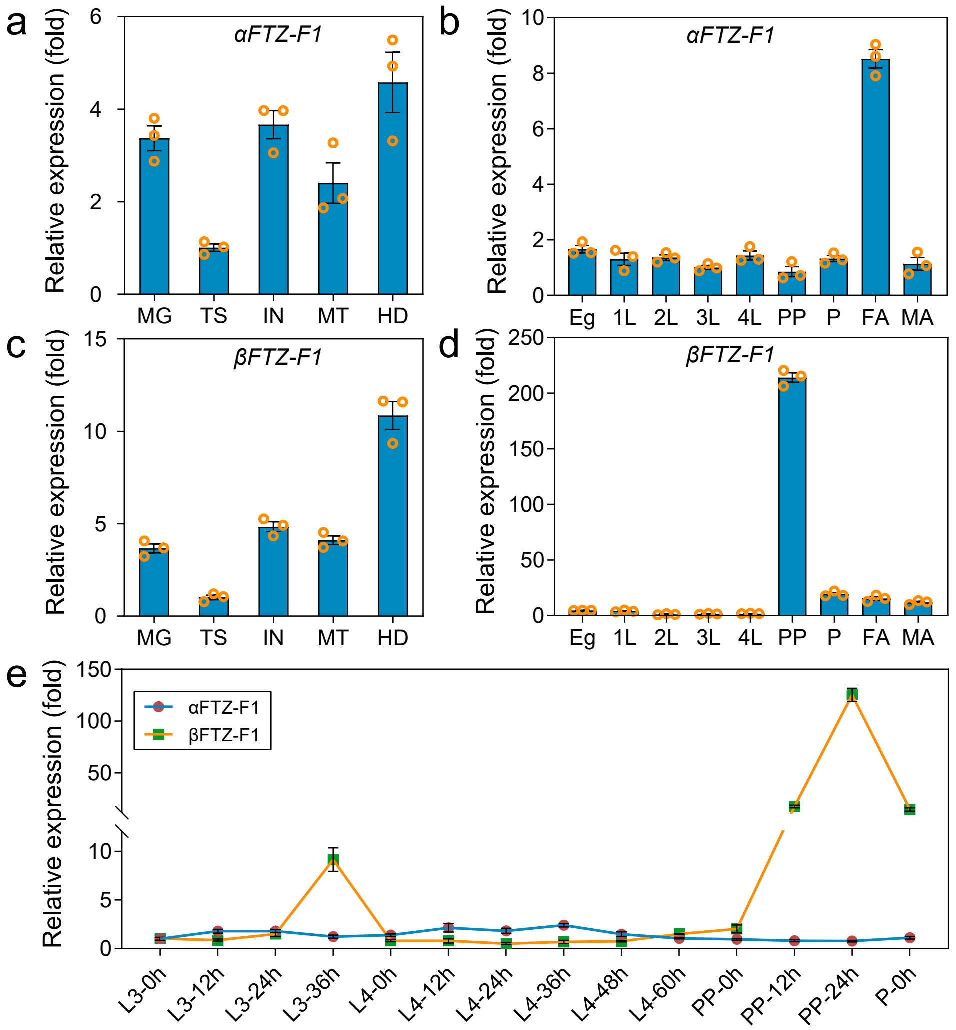

Supplementary Figure 2. Spatio-temporal expression profiles of the FTZ-F1 in *P. xylostella*. a-d The relative expression of αFTZ-F1 in different tissues (**a**) and developmental stages (**b**). The relative expression of βFTZ-F1 in different tissues (**c**) and developmental stages (**d**). **e** The relative expression of αFTZ-F1 and βFTZ-F1 from third-instar larvae to pupae. Data were normalized to the expression of the *RPL32* gene, and the expression level (fold) was calculated based on the value of the lowest expression. The values shown are the means and the corresponding standard error (SEM). Abbreviation: Eg (egg); HD (head); IN (integument); MG (midgut); TS (testis) and MT (Malpighian tubules); L1-L4 (first- to fourth-instar larvae); PP (prepupae); P (pupae); MA (male adults); FA (female adults). Source data are provided as a Source Data file.

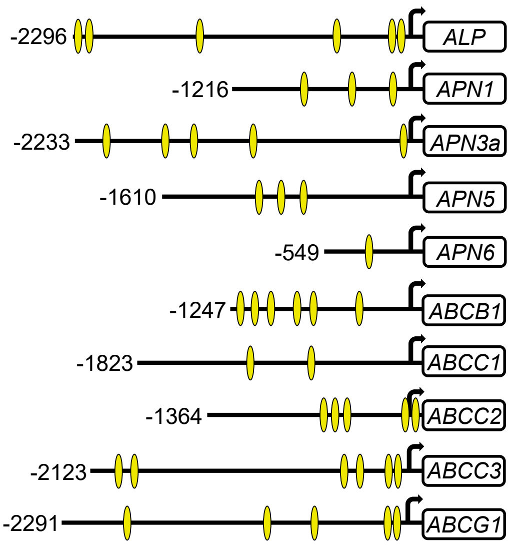

Supplementary Figure 3. Potential FTZ-F1 binding sites (FBSs) predicted in Cry1Ac toxin receptor and non-receptor paralogous genes are depicted by yellow ellipses.

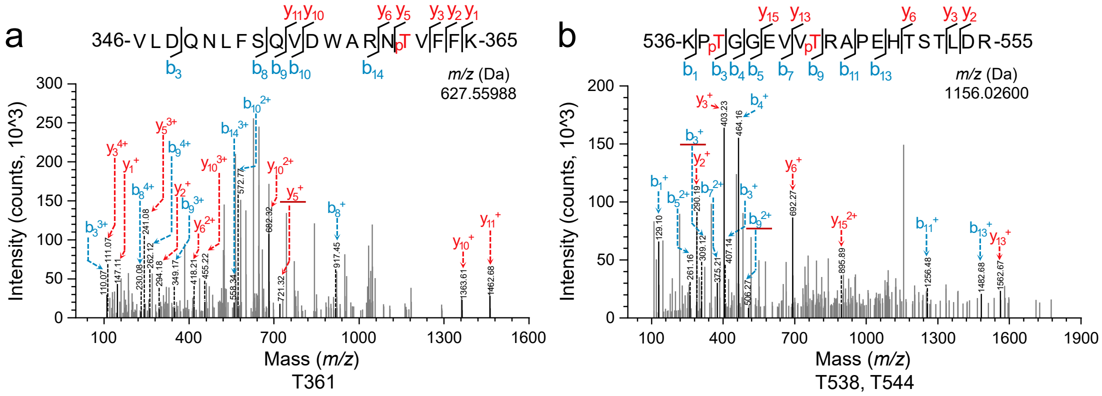

Supplementary Figure 4. LC-MS/MS analysis of wild type FTZ-F1. a, b The phosphorylation sites T361 (**a**), T538 and T544 (**b**) are identified by LC-MS/MS. The identified phosphorylation sites, underlined in red, are represented by a red letter and subscripted p in the precursor ions.

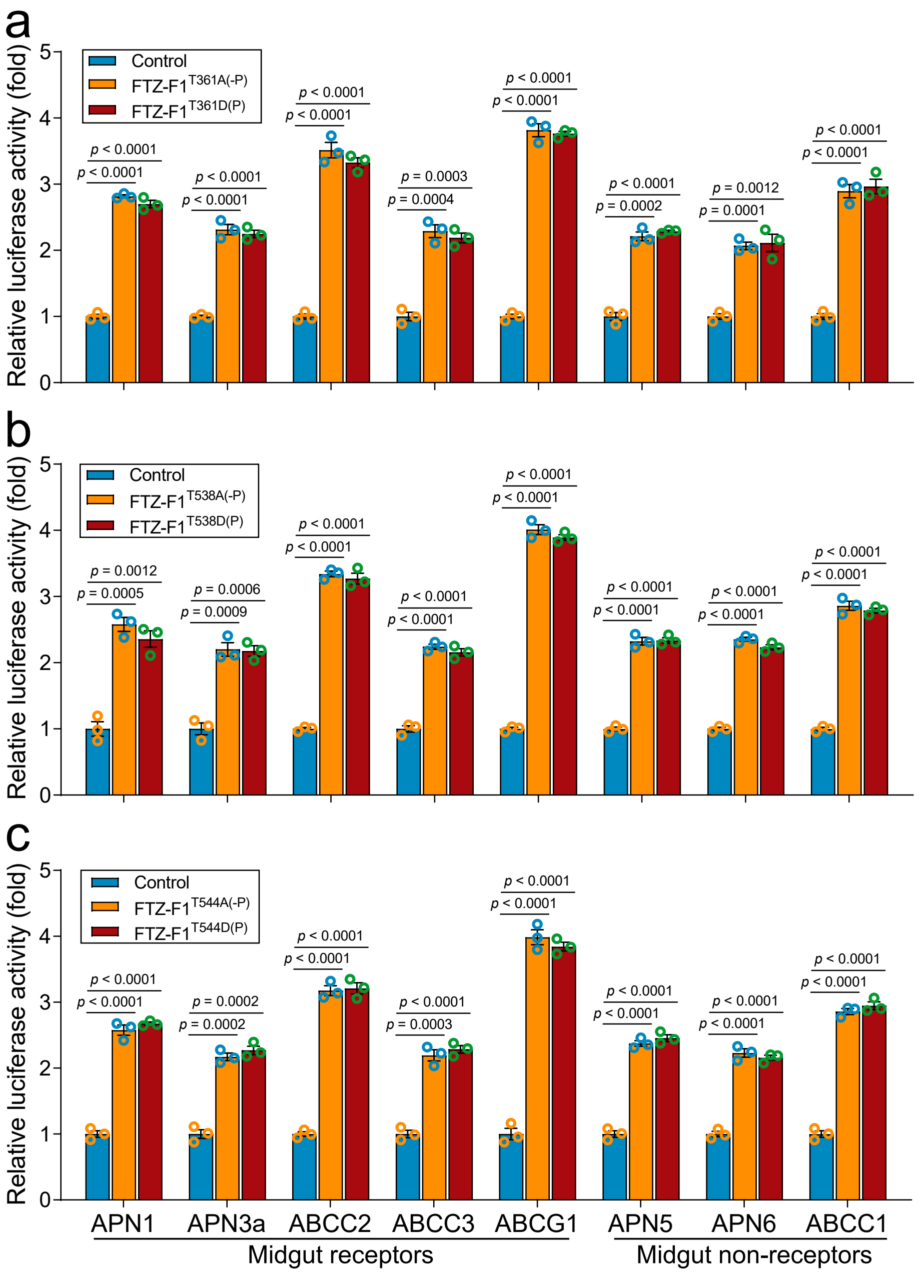

Supplementary Figure 5. The regulatory effect of three identified phosphorylation sites on midgut genes. (a) T361, (b) T538 and (c) T544. A substitutes T mimicking dephosphorylation, D substitutes T mimicking sustained phosphorylation. The empty pAc5.1 vector was used as control. The relative luciferase activity (fold) was standardized by the value of the control. Data are presented as mean values ± SEM (n = 3), ns, not significant, *p*-values are shown. One-way ANOVA with Tukey's test was used for comparison. Source data are provided as a Source Data file.

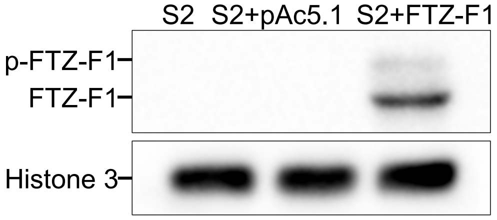

Supplementary Figure 6. Phosphorylation and non-phosphorylation levels of FTZ-F1 in the S2 cell line. Nucleoproteins were extracted from untransfected S2 cells and cells transfected with empty vector pAc5.1 or FTZ-F1. Histone 3 was used as an internal loading control. Source data are provided as a Source Data file.

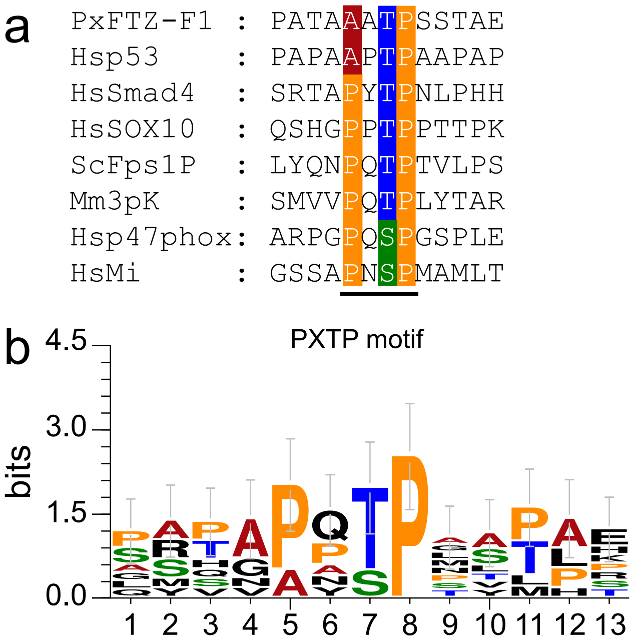

Supplementary Figure 7. The MAPK consensus target sequences in the PXTP motif. a All PXTP motifs are derived from different proteins, full-length amino acid sequences of genes were retrieved from the GenBank database. The protein p53 (NP_001119590.1), Smad4 (NP_005350.1), SOX10 (NP_008872.1), p47phox (AAB95193.1) and Mi (NP_001341533.1) are derived from *Homo sapiens*, the protein Fps1P (NP_013057.1) and 3pK (NP_849238.1) are derived from *Saccharomyces cerevisiae* and *Mus musculus*, respectively. **b** WebLogo plot displays amino acid conservation in the PXTP motifs.

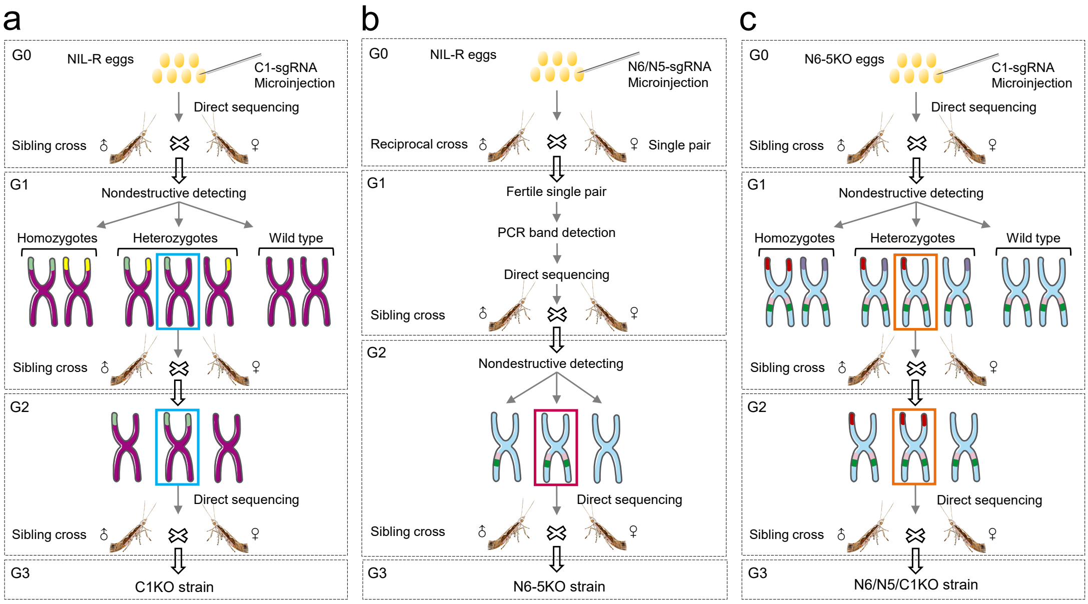

Supplementary Figure 8. Schematic diagram of the detailed crossing scheme used to obtain the homozygous single C1KO, double N6-5KO and triple C1/N6/N5KO knockout strains. a A mixture of Cas9 protein and sgRNA targeting *ABCC1* gene was microinjected into eggs from the resistant NIL-R strain. CRISPR/Cas9-induced gene mutations were identified by direct sequencing, and different mutant genotypes were further detected by direct sequencing and TA cloning in G1 individuals. For these genotypes, patches with different colors on chromosomes denote the regions with a disrupted *ABCC1* gene. **b** A mixture of Cas9 protein and two sgRNAs targeting either *APN6* or *APN5* was co-injected into eggs from the resistant NIL-R strain. Single-pair reciprocal crosses between G0 and NIL-R were performed to generate G1 progeny. The single pair group with mutagenesis was reared to pupation and the moths were sib-crossed with each other to generate G2 progeny. The adjacent patches with different colors on chromosomes indicate mutagenesis of *APN6* and *APN5* genes. **c** A mixture of Cas9 protein and sgRNA targeting *ABCC1* was injected into eggs from the double gene knockout strain (N6-5KO) to generate the triple mutant strain (C1/N6/N5KO).

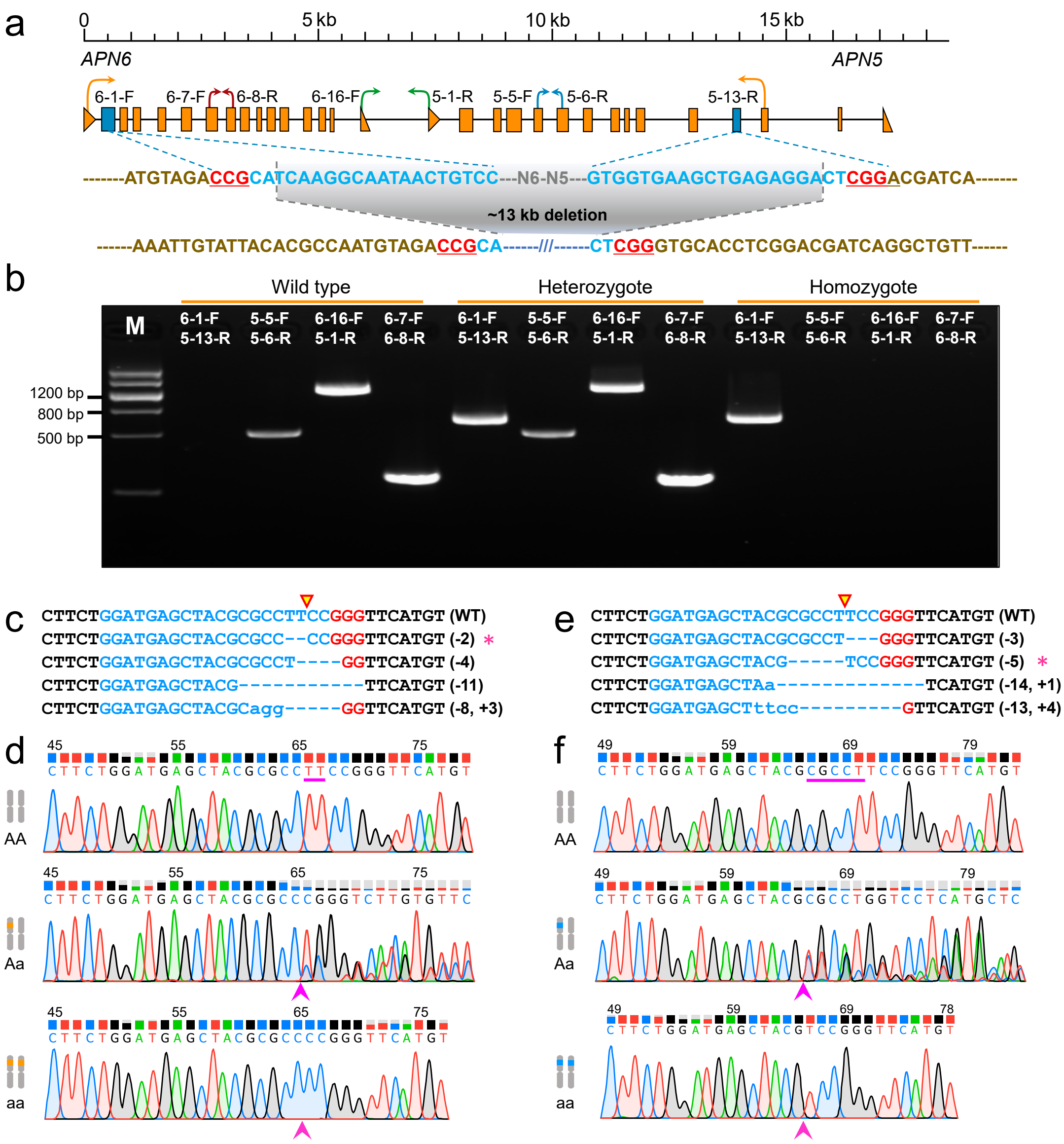

Supplementary Figure 9. CRISPR/Cas9-mediated knockout of non-receptor genes. a CRISPR/Cas9-based double gene knockout of *APN6* and *APN5* in *P. xylostella*. The genomic structure of *APN6* and *APN5*, the sgRNA sequences are highlighted in blue text and the protospacer adjacent motif (PAM) sequences by underlined red text. The disrupted sequences are shown in the gray dashed box. **b** Genotype detection of the double gene mutation in *APN6* and *APN5* locus according to the banding profile of PCR products from the four primer pairs marked in (**a**). **c, e** Different types of indel mutations surrounding the sgRNA target sites of *ABCC1* in G1 individuals identified by TA cloning and sequencing of PCR products based on parental NIL-R (**c**) and double mutant N6-5KO strains (**e**), respectively. The deleted bases are represented as dashes, and the inserted bases are shown as lowercase letters. The numbers of inserted or deleted bases are displayed at the right of each allele (+,

insertion; –, deletion). Asterisks indicated the screened monoallelic mutants with sufficient individuals used for further sib-mated to generate G2 progeny. **d, f** Representative chromatograms of direct sequencing of the PCR products from wild types (upper graph), heterozygotes (middle graph), and homozygotes (lower graph) of *ABCC1* in G2 larvae based on parental NIL-R (**d**) and double mutant N6-5KO strains (**f**), respectively. The 2-bp (TT) deletion and 5-bp deletion (CGCCT) in exon 2 of the *ABCC1* gene are highlighted by pink lines and arrows. Source data are provided as a Source Data file.

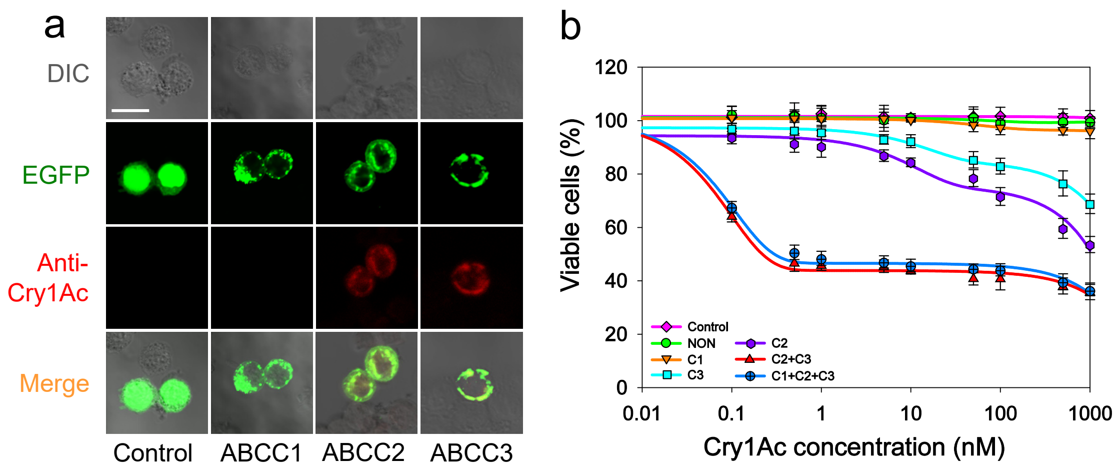

Supplementary Figure 10. Expression of recombinant EGFP-ABCC1-3 proteins in insect Sf9 cells. a Immunofluorescence detection of Cry1Ac binding to Sf9 cells expressing EGFP-ABCC1-3 fusion proteins. Gray panels: differential interference contrast (DIC) microscopy views; green panels: fluorescence of EGFP-fusion proteins; red panels: Cry1Ac-binding fluorescent signal with rabbit polyclonal anti-Cry1Ac primary antibody and goat anti-rabbit secondary antibody conjugated with red fluorescence Alexa Fluor 555; superimposed panels: merged images from both the green and red fluorescent channels. The scale bar is 15 μm. **b** Susceptibility to different concentration gradients of Cry1Ac toxin (0.1 nm-1 μm) in untransfected Sf9 cells (Control), Sf9 cells expressed with an empty expression vector (NON), ABCC1-3-expressing Sf9 cell, and Sf9 cell with simultaneous expression of multiple proteins (both ABCC2 and ACCC3, as well as ABCC1-3). Cell viability was detected by CCK-8 assay after 24 h incubated with different concentrations of Cry1Ac toxin. The data are indicated as mean ± SEM. Source data are provided as a Source Data file.

Supplementary Table 1. CRISPR/Cas9-induced single and triple knockout in *P. xylostella*

Original strains	G0				G1		G2		Mutant strains
	Eggs[#]	Hatched (%)	Adults (%)	Mutant (%)[*]	Pupae	Genotypes (%)[‡]	Sib-cross[†]	Homozygous (%)[¶]	
NIL-R	260	168/260 (65)	91/168 (54)	66/91 (73)	109	52/109 (48)	—	—	C1KO
						17/109 (16)	—	—	
						40/109 (37)	-2 (22)√	27 (15♂, 12♀)	
							-4 (9)	—	
							-11 (7)	—	
							-8, +3 (2)	—	
N6-5KO	280	144/280 (51)	68/144 (47)	48/68 (71)	90	49/90 (54)	—	—	N6/N5/C1KO
						11/90 (12)	—	—	
						30/90 (33)	-5 (20)√	18 (10♂, 8♀)	
							-3 (6)	—	
							-14, +1 (3)	—	
							-13, +4 (1)	—	

[#]A total of 260 and 280 fresh preblastoderm eggs from the resistant NIL-R and double-knockout N6-5KO strains were respectively collected for microinjection.

[*]Nondestructive genotyping of indel mutations surrounding the sgRNA target site was performed by PCR method, and 73% (66/91) and 71% (48/68) individuals showed high site-specific mutagenesis efficiency in CIKO and N6/N5/C1KO groups in G0 offspring, respectively.

[‡]A total of 109 and 90 pupae of G1 were obtained for genotyping. For C1KO and N6/N5/C1KO groups, 48% (52/109) and 54% (49/90), WT; 16% (17/109) and 12% (11/90), biallelic heterozygous and homozygous; 37% (40/109) and 33% (30/90), monoallelic heterozygous.

[†]As for monoallelic heterozygous, four genotypes were obtained in both groups, and the largest number of individuals (-2 and -5 bp) in C1KO and N6/N5/C1KO groups with about 1:1 sex ratio were sib-crossed with each other to further generate G2 progeny.

[¶]27 and 18 homozygotes were determined by direct sequencing.

Supplementary Table 2. CRISPR/Cas9-mediated double-gene knockout of *APN5* and *APN6* in *P. xylostella*

Original strain	G0				G1		G2		Mutant strain
	Eggs	Hatched (%)[#]	Adults (%)[&]	Reciprocal crosses*	Fertile progeny (%)[†]	Heterozygous (%)[‡]	Pupae	Homozygous (%)[¶]	
NIL-R	430	130/430 (30)	62/130 (48)	25 × 25	22/50 (44)	28/65 (43)	98	20/98 (20)	N6-5KO

[#]Among the injected eggs, approximately 30% (130/430) hatched to larvae.

[&]Among the hatched larval, about 48% (62/130) of the larvae developed into adults in G0.

*25 single-pair reciprocal crosses between the G0 progeny and the resistant NIL-R strain were performed to produce G1 progeny.

[†]Among the 50 single pairs, 22 single pairs produce fertile progeny and two of the 22 single pairs exhibited mutagenesis in *APN5* and *APN6* locus by the specific PCR amplification.

[‡]The other larvae from the two pair groups with the positive mutation were reared to pupation, the gDNA samples from 65 exuviates of the fourth-instar larvae in one of the two pair groups were used for nondestructive detection, and 28 of them were heterozygotes.

[¶]20 homozygous individuals were ascertained by direct sequencing using the gDNA samples from 98 exuviates of the final fourth-instar larvae.

Supplementary Table 3. Resistance to Cry1Ac toxin in larvae from DBM1Ac-S, NIL-R and mutant strains of *P. xylostella*

Strains	N*	LC_{50} (95% CL)†	Slope ± SE	χ^2(df)‡	RR§
DBM1Ac-S	210	0.77 (0.59 - 0.97)	2.11 ± 0.25	2.01(5)	1.00
NIL-R	210	3979.91 (2863.76-5896.18)	2.35 ± 0.29	5.83(5)	5168.71
C1KO	210	3933.44 (2801.70-5954.39)	2.16 ± 0.27	5.65(5)	5108.36
N6-5KO	210	3876.23 (3031.36-5136.54)	2.12 ± 0.27	3.37(5)	5034.06
N6/N5/C1KO	210	3824.53 (2734.72-5729.09)	2.20 ± 0.27	5.65(5)	4966.92

*Number of larvae tested (larvae of the control group not included).

†Concentration of Cry1Ac toxin (mg/L) killing 50% of larvae and its 95% confidence limits (CL).

‡The value of chi-square and degrees of freedom (df) were calculated by Polo Plus 2.0.

§RR: Resistance ratio (RR) calculated by LC_{50} of resistant or mutant divided by LC_{50} of DBM1Ac-S.

Supplementary Table 4. Primers used in this study

Purpose	Gene	Primer name	Primer sequence (5′-3′)	PCR product size (bp)	Positions
Promoter cloning	APN1	APN1-F	AGCTTCCTCTCAGTGTTCAC	1785	-1216/+569
		APN1-R	TCTGTTTGTAGGTTGCCCC		
	APN3a	APN3a-F	GCAGTATGGGCAGTAAGAA	2339	-2233/+106
		APN3a-R	CATCGGTGAAAATGGTGTT		
	APN5	APN5-F	CGCCACGAAGGATGG	1665	-1610/+55
		APN5-R	CCCAAGCCAGGGCAAGAAG		
	APN6	APN6-F	ATCGTCATTGTTTGCTCCT	637	-549/+88
		APN6-R	TTTCATTTCTATCCGTCCC		
	ABCC1	ABCC1-F	CGTAGTGTCTTTCCCTCTG	1897	-1823/+74
		ABCC1-R	TTGTTCGTCTTCTTGTCCC		
	ABCC2	ABCC2-F	ATTTCCCGATAGGCTGTGA	1482	-1364/+118
		ABCC2-R	GGCTTGCCCTTCTTCACCT		
	ABCC3	ABCC3-F	AATACCTCACCTGCCCACC	2148	-2123/+25
		ABCC3-R	CATCCTCCGCAACCTTCAC		
	ABCG1	ABCG1-F	ATGTCGATGTCGGTGGTT	2326	-2291/+35
		ABCG1-R	ATCAGAGGCTCCTGCTCC		
	ALP	ALP-F	TTGGCTGAACTGATTTTAATG	2393	-2296/+97
		ALP-R	CCTTCTTGGGCTTCGGTGT		
	ABCB1	ABCB1-F	TTAGTTACATAGTTACCGACAC	1272	-1247/+25
		ABCB1-R	TTGAGTCTTCGTTGAAATT		
pGL4.10 recombinant†	APN1	P(-1216/-1)-F	AGCTTCCTCTCAGTGTTCAC	1216	-1216/-1
		P(-829/-1)-F	GTAACCTCATCACTACCTAACAAA	829	-829/-1
		P(-757/-1)-F	TGTGTCGTCAAGCAATCAAGTCAG	757	-757/-1
		P(-500/-1)-F	TATGCCGTGGTGAAAGGACAAC	500	-500/-1
		P(-469/-1)-F	ATAGTGTCAGACACTGACTG	469	-469/-1
		P(-241/-1)-F	GTTCAGTAAGTACTATTAAC	241	-241/-1

	P(-200/-1)-F	AACTAATAACGGGCTTTAATATGGA	200	-200/-1
	Promoter-R	TTTCTCGCCAGTATAAAGCTA	—	—
APN3a	P(-2233/-1)-F	GCAGTATGGGCAGTAAGAA	2233	-2233/-1
	P(-1700/-1)-F	GCTGTCGCCAAAATAACAAGGT	1700	-1700/-1
	P(-1518/-1)-F	CCTTATAATTATGATAATCT	1518	-1518/-1
	P(-1130/-1)-F	CCACGTAATTTGCAGCGACA	1130	-1130/-1
	P(-1091/-1)-F	ACCGTCGCTCGTCTATCAAA	1091	-1091/-1
	P(-160/-1)-F	TTTCATAAATTGCGAGGCTA	160	-160/-1
	P(-129/-1)-F	CTTACTTCAGTATTCGTTATTTG	129	-129/-1
	Promoter-R	TTCATTACTTATCTAATTTATTTTAG	—	—
APN5	P(-1610/-1)-F	CGCCACGAAGGATGG	1610	-1610/-1
	P(-1528/-1)-F	AGTTACGTTGACATTGCCGT	1528	-1528/-1
	P(-1418/-1)-F	AACGTACCCTACATAGGATAT	1418	-1418/-1
	P(-1314/-1)-F	CATTCAATTTATACTGTTGAATTAT	1314	-1314/-1
	P(-1200/-1)-F	CAATCTGAACCACCCAGC	1200	-1200/-1
	P(-1170/-1)-F	CCTATCATAATATTTCATCTAGC	1170	-1170/-1
	P(-1141/-1)-F	CATAGCATGCATAACTGCATTT	1141	-1141/-1
	P(-1119/-1)-F	CCCACCTAGCCTTAGGGTA	1119	-1119/-1
	P(-1100/-1)-F	TACTGGTTGAGCTAATATTG	1100	-1100/-1
	P(-1048/-1)-F	TTTATAGTATTAAGTGGAGGTTAAG	1048	-1048/-1
	P(-900/-1)-F	GATAGAGAAGAGTGGAGGG	900	-900/-1
	P(-776/-1)-F	TAAGCTTGGACCATTTCCCA	776	-776/-1
	Promoter-R	GCTTCTACAAGAAAATATTATTTC	—	—
APN6	P(-549/-1)-F	ATCGTCATTGTTTGCTCCT	549	-549/-1
	P(-412/-1)-F	TTGCATTATTCTTATTTGAAGGAT	412	-412/-1
	P(-380/-1)-F	GTAAAATTCCATGTAACATTATGA	380	-380/-1
	P(-313/-1)-F	ACGTAATAATCTCTTTATCAGTAT	313	-313/-1
	P(-289/-1)-F	CATACTACTAAATAATAAGGGCA	289	-289/-1
	P(-266/-1)-F	ATTACCATACAGTCTTAATCGGC	266	-266/-1

	P(-244/-1)-F	CACTAATATCGATAACTATAGAG	244	-244/-1
	P(-232/-1)-F	TAACTATAGAGATAAGATAGGTAAC	232	-232/-1
	P(-143/-1)-F	GCTGATCAGGTAAGTACGTT	143	-143/-1
	P(-59/-1)-F	TTAAACCATATCATAAAGTGTCTT	59	-59/-1
	Promoter-R	CATGCCTGTAATATTTTAAATTTA	—	—
ABCC1	P(-1823/-1)-F	CGTAGTGTCTTTCCCTCTG	1823	-1823/-1
	P(-1149/-1)-F	AATGCGCCATTCATTCTCAT	1149	-1149/-1
	P(-1095/-1)-F	AACAAGTCTAAGTACCTATGTAC	1095	-1095/-1
	P(-1039/-1)-F	TGCAGTTTCGCTTTAGCCAG	1039	-1039/-1
	P(-989/-1)-F	AGTTGCGGACCAGTTCTACA	989	-989/-1
	P(-910/-1)-F	AAATAAGCAAAAGCGCATGA	910	-910/-1
	P(-839/-1)-F	TGTGAACGCAGTACAGTCAC	839	-839/-1
	P(-823/-1)-F	TCACGGATAGCTAAGAGGAT	823	-823/-1
	P(-805/-1)-F	ATATTTCAGGTACCTACTTACTT	805	-805/-1
	P(-785/-1)-F	CTTATACAATATTTATCGATATGTT	785	-785/-1
	P(-717/-1)-F	TTGTAAATAATCTGCCATTATCAA	717	-717/-1
	Promoter-R	TTTGAATGTGTTTTTGTAACTACAA	—	—
ABCC2	P(-1364/-1)-F	ATTTCCCGATAGGCTGTGAA	1364	-1364/-1
	P(-653/-1)-F	TTAACGAATACCTATGTCCTTG	653	-653/-1
	P(-609/-1)-F	TGGATGTATGTATGTATGTATG	609	-609/-1
	P(-547/-1)-F	GATCAGCTGTCTTGCAGTG	547	-547/-1
	P(-497/-1)-F	GGTGTGCCATAGGACTGCAA	497	-497/-1
	P(-128/-1)-F	AATATTGACTGTTGAGTGAGAC	128	-128/-1
	P(-100/-1)-F	TTGTTGTGCAACCTGTCCTG	100	-100/-1
	Promoter-R	TTCGACACATGTAGTTTGCAC	—	—
ABCC3	P(-2123/-1)-F	AATACCTCACCTGCCCACC	2123	-2123/-1
	P(-1998/-1)-F	CGCTATAAATCATATTTCGCTT	1998	-1998/-1
	P(-1959/-1)-F	CTGAATTGTACTGCTCTGTTTG	1959	-1959/-1
	P(-1857/-1)-F	ATCAGGCATGTACCAACGAT	1857	-1857/-1

		P(-532/-1)-F	AAATAGACGGTACAATTCGCG	532	-532/-1
		P(-496/-1)-F	GTAATCTACCAGACTACCTC	496	-496/-1
		P(-150/-1)-F	CCGTTTGATAAGACATTCGAG	150	-150/-1
		P(-130/-1)-F	GTGTGGCGCGGGCACCA	130	-130/-1
		P(-110/-1)-F	AACATGTGGCTTGTGTTTGTC	110	-110/-1
		Promoter-R	TCTTATATCACTATTAATTAATTAAT	—	—
	ABCG1	P(-2291/-1)-F	ATGTCGATGTCGGTGGTTTAGC	2291	-2291/-1
		P(-1906/-1)-F	TTTATTTGCATACGTAGCATTGA	1906	-1906/-1
		P(-1871/-1)-F	ACACTGCCAATTATTGAACCAT	1871	-1871/-1
		P(-1034/-1)-F	AAATATGTTCATGGGAATGGT	1028	-1028/-1
		P(-993/-1)-F	AAGTAACCGATTGAATAAACGAA	993	-993/-1
		P(-716/-1)-F	AATTAAATACGAATCAAGGTTTGT	716	-716/-1
		P(-682/-1)-F	TCGCAATGTTATTGTCAAATAGA	682	-682/-1
		P(-222/-1)-F	TGCTAGATGAAACACCTCCC	222	-222/-1
		P(-152/-1)-F	CGCTCGAGTCAGTAGGCT	152	-152/-1
		P(-100/-1)-F	CGATACCAAAGACAAACCCG	100	-100/-1
		Promoter-R	TTTGTAAAAAAAAAAACACTAATCG	—	—
	ALP	P(-2296/-1)-F	TTGGCTGAACTGATTTTAATGAA	2296	-2296/-1
		Promoter-R	GACTGACTCGCAGACGC	—	—
	ABCB1	P(-1247/-1)-F	TTAGTTACATAGTTACCGACACAC	1247	-1247/-1
		Promoter-R	TTTGACTGTACGTTTTTATATCTT	—	—
TF cloning	αFTZ-F1	αFTZ-F1-F	AGGTGGTGAACTTATTGCGTATC	1773	-68/+1705
		αFTZ-F1-R	CTAACCGCCCGTCTCATTC		
	βFTZ-F1	βFTZ-F1-F	CCGCCAGCAGCAGATAGT	1957	-124/+1833
		βFTZ-F1-R	CCGGCGCTCAGCAATAAG		
	PREB	PREB-F	ACTTGTGTGTATGCGAAAAGTGA	1445	-76/+1369
		PREB-R	CCCCGAGGTACTTACAGTGAAAT		
	RB1CC1	RB1CC1-F	AAAGCGGCCATCAAATTGCC	4048	-23/+4025
		RB1CC1-R	TTCGGATTGTGTTACCGGGG		

pAc5.1-TF‡	αFTZ-F1	In-αFTZ-F1-F	ATGCACGAGGAGGCCCCA	1671	+1/+1671
		In-αFTZ-F1-R	ACATAATCTGTCAAGTGTGGA		
	βFTZ-F1	In-βFTZ-F1-F	ATGACGATGGATCAGCAAAC	1683	+1/+1683
		In-βFTZ-F1-R	ACATAATCTGTCAAGTGTGGA		
	PREB	In-PREB-F	ATGGCACCAAATCGAAGCGA	1296	+1/1296
		In-PREB-R	AATGCCGAAATACGAGCAGAG		
	RB1CC1	In-RB1CC1-F	ATGCTGTATGTGTTTCATGTGG	3960	+1/3960
		In-RB1CC1-R	GGGGGTCGCTTCTTCAGC		
pGADT7-FTZ-F1§	βFTZ-F1	FTZ-F1-Y1H-F	ATGACGATGGATCAGCAA	1683	+1/+1683
		FTZ-F1-Y1H-R	ACATAATCTGTCAAGTGTGGA		
pie2-EGFP-N1-FTZ-F1*	βFTZ-F1	FTZ-F1-EGFP-F	CTCAAGCTTCGAATTGCCACCATGACGATGGATCAGCAAACTGG	1683	+1/+1683
		FTZ-F1-EGFP-R	GGCGACCGGTGGATCTCCTCCTCCTCCACATAATCTGTCAAGTGTGGACG		
qPCR analysis	APN1	qAPN1-F	TCACTGAGTCCCATCCCA	157	+58/+214
		qAPN1-R	TGCCAGACGGCACATTTT		
	APN3a	qAPN3a-F	GGCTACCGTTGGCTACAC	100	+2190/+2299
		qAPN3a-R	GCAGACATTCCTCCACTCC		
	APN5	qAPN5-F	GGACGATCAGGCTGTTAA	160	+2412/+2571
		qAPN5-R	TTCCCGAATCTGGTTGTG		
	APN6	qAPN6-F	CAGCAGGGACGGATAGAA	146	+65/+210
		qAPN6-R	ATCGGTAGCAACGAAGTTAA		
	ALP	qALP-F	GCACACACCATGACCGTAGCAG	169	+1207/+1375
		qALP-R	GGCTCTTCGTGACATCG		
	ABCB1	qABCB1-F	AGCGAAAGGAGATTGATAGG	157	+942/+1098
		qABCB1-R	GTAATAAACTGGAAACCGAAC		
	ABCC1	qABCC1-F	GGTGGTGCTCATCTGCTACCTCAT	165	+696/+860
		qABCC1-R	ATCCTGACACGCTCATCGGTTTT		
	ABCC2	qABCC2-F	AGTCTTGGCACGCAAACGG	103	+2401/+2503
		qABCC2-R	CGAACAGACGCATGAAGGACAT		

	ABCC3	qABCC3-F	TCAACCGCTTCACCAAGGACAT	111	+2606/+2716
		qABCC3-R	CGGCGTTCAGCACCAGGAT		
	ABCG1	qABCG1-F	ATCTGGTGTTTCAGGCTTTAGTC	118	+545/+662
		qABCG1-R	ATCACGGTGTTCTGGCATTT		
	αFTZ-F1	qαFTZ-F1-F	GCCAAAGAGCGAAATGCGAG	74	+60/+133
		qαFTZ-F1-R	TCTCGGTCATTTTGGGACCG		
	βFTZ-F1	qβFTZ-F1-F	CAAACTGGCCTCATGTCCCT	119	+16/+134
		qβFTZ-F1-R	TGGTAAGCTGCTCCTTGTGG		
	FTZ-F1	qFTZ-F1-F	CAGAACTCGCTCTTCGGACT	122	+1026/+1148
		qFTZ-F1-R	GTTTCTCGCCCAATCGACCT		
	MAP4K4	qMAP4K4-F	CATCAACTGGCTCCGTCTG	188	+7/+215
		qMAP4K4-R	TCATCTTCGGTGACATCCATC		
	RPL32	qL32-F	CCAATTTACCGCCCTACC	120	—
		qL32-R	TACCCTGTTGTCAATACCTCT		
dsRNA synthesis[#]	FTZ-F1	dsFTZ-F1-F	CTCCAGAACAAGCTCGCTGA	377	+1285/+1661
		dsFTZ-F1-R	TCAAGTGTGGACGTGTGCTC		
	MAP4K4	dsMAP4K4-F	T7-GCCCGAGATACGCAAATACA	582	+2739/+3274
		dsMAP4K4-R	T7-CCGAGCCATAGATCACTTTCA		
	EGFP	dsEGFP-F	T7-CCACAAGTTCAGCGTGTCCG	469	—
		dsEGFP-R	T7-AAGTTCACCTTGATGCCGTTC		
CRISPR sgRNA[&]	ABCC1	CRI-C1-F	*GAAATTAATACGACTCACTATAGG***GGATGAGCTACGCGCCTTCC**GTTTTAGAGCTAGAAATAGC	124	—
		CRI-C1-R	AAAAGCACCGACTCGGTGCCACTTTTTCAAGTTGATAACGGACTAGCCTTATTTTAACTTGCTATTTCTAGCTCTAAAAC		
	APN6	CRI-N6-F	*GAAATTAATACGACTCACTATAGG***GGACAGTTATTGCCTTGATG**GTTTTAGAGCTAGAAATAGC	124	—
		CRI-N6-R	AAAAGCACCGACTCGGTGCCACTTTTTCAAGTTGATAACGGACTAGCCTTATTTTAACTTGCTATTTCTAGCTCTAAAAC		
	APN5	CRI-N5-F	*GAAATTAATACGACTCACTATAGG***GTGGTGAAGCTGAGAGGACT**GTTTTAGAGCTAGAAATAGC	124	—

		CRI-N5-R	AAAAGCACCGACTCGGTGCCACTTTTTCAAGTTGATAACGGACTA GCCTTATTTTAACTTGCTATTTCTAGCTCTAAAAC		
Identification of genes knockout¶	ABCC1	C1-F	GATTTAAGTATTTCTTTATTCCAAC	300	—
		C1-R	ACAACTCCAAATATCATCTAC		
	APN6-5	6-1-F	CCAGCCTACAGTTATAATGGG	701	—
		5-13-R	CAGTCAGTACCATTTCCAAGTAC		
	APN6	6-7-F	ATCAGCTATTCTAAAGGAGCTGCTT	361	—
		6-8-R	CCTCATAACCATTCAGTGTTCCATC		
	APN6-5	6-16-F	ATGGAGCAGCACAAATTGTTG	1558	—
		5-1-R	GCTAGAGCAAGAAGAACTGGC		
	APN5	5-5-F	GGACACTTCAGTGGCATCAGCTACT	505	—
		5-6-F	CATTCGTGAACTGAGTGAGAGCG		
Full-length cDNA cloning	ABCC1	C1-F-F	ATGGTGAGTGGTAATAAGGACCCGT	4164	+1/+4161
		C1-F-R	TTCTATCACTCCGCCATTATTAAGG		
	ABCC2	C2-F-F	ATGGAAAACGGAAGCGGAGC	4041	+1/+4038
		C2-F-R	TTGAGGATGGTCGTCGAAGTATTTC		
	ABCC3	C3-F-F	ATGGGGGTGAAGGTTGCGGA	4044	+1/+4041
		C3-F-R	CCTAGCGTTCTCTTTCATATTGCTT		
Heterologous expression¥	ABCC1	C1-EXP-F	*CCC*AAGCCTATGGTGAGTGGTAATAAGGACCCGT	4182	—
		C1-EXP-R	*CTA*GCTAGCTTCTATCACTCCGCCATTATTAAGG		
	ABCC2	C2-EXP-F	*CCC*AAGCCTATGGAAAACGGAAGCGGAGC	5059	—
		C2-EXP-R	*CTA*GCTAGCTTGAGGATGGTCGTCGAAGTATTTC		
	ABCC3	C3-EXP-F	*CCC*AAGCCTATGGGGGTGAAGGTTGCGGA	4062	—
		C3-EXP-R	*CTA*GCTAGCCCTAGCGTTCTCTTTCATATTGCTT		

†A 15-bp sequence (TGGCCTAACTGGCCG) is added to 5′-ends of the forward primers of pGL4.10 recombinants; A 15-bp sequence (CGCCGAGGCCAGATC) is added to 5′-ends of the reverse primers of pGL4.10 recombinants.

‡A 15-bp sequence (AGACCCCGGATCGGG) is added to 5′-ends of forward primers of pAc5.1-TF recombinant plasmids; A 15-bp sequence (GCCCTCTAGACTCGA) is add to 5′-ends of reverse primers of pAc5.1-TF recombinant plasmids.

[§]A 15-bp sequence (GGAGGCCAGTGAATT) is added to the 5′-end of forward primer of pGADT7-FTZ-F1 recombinant plasmid; A 15-bp sequence (TCATCTGCAGCTCGA) is added to the 5′-end of the reverse primer of pGADT7-FTZ-F1 recombinant plasmid.

[*]A 15-bp sequence (CTCAAGCTTCGAATT) is added to the 5′-end of forward primer of pie2-EGFP-N1-FTZ-F1 recombinant plasmid, before the ATG initiation codon Kozak consensus sequences (GCCACC) were included to increase the translation efficiency. A 15-bp sequence (GGCGACCGGTGGATC) and linker sequences (TCCTCCTCCTCC) encoding four tryptophan are added to the 5′-end of reverse primer of pie2-EGFP-N1-FTZ-F1 recombinant plasmid.

[#]The T7 RNA polymerase promoter sequence (TAATACGACTCACTATAGGGAGA) is added to 5′-ends of forward and reverse primers to synthesize dsRNA template.

[&]A specific oligonucleotide encoding a T7 polymerase-binding site (italicized) and the sgRNA target sequence (underlined and bold) of *ABCC1*and *APN5/6* were designed as the forward primer CRISPR-F, and a common oligonucleotide encoding the remaining sequences of CRISPR-F were designed as the reverse primer CRISPR-R.

[¶]To further validate the accurate information of the indel sequences, we amplified the genome DNA fragment (approximately 300 bp) of *ABCC1* flanking CRISPR target site by using the exuviate gDNA of fourth-instar *P. xylostella* larvae as a template with the specific sequencing primers, and the PCR products of the mutants were ligated into *pEASY*-T1 cloning vector and sequenced for validation of the genomic mutated events. Additionally, to verify the genotypes of the double-knockout individuals in N6-5KO strains, four DNA products of different sizes were amplified by the PCR method with four pairs of primers between *APN6* and *APN5* locus, respectively. For primer names, the first number shows the specific gene (6 and 5 represent for *APN6* and *APN5* gene), and the second number indicates as exon. A 701 bp genome DNA fragment was amplified with the primers 6-1-F/5-13-R if *APN6* and *APN5* genes were simultaneously deleted. The primer pairs of 6-7-F/6-8-R, 6-16-F/5-1-R, and 5-5-F/5-6-R were used to respectively amplify the gDNA fragments of 361, 1558 and 505 bp to detect the double-mutant individuals are homozygous or heterozygous in N6-5KO strain.

[¥]Using restriction enzymes *Hind*III and *Nhe*I to cut the pie2-EGFP-N1 expression vector and generate a linearized vector, then the protective bases CCC (italicized) and restriction endonuclease *Hind*III (underlined) were added before the upstream primers of ABCC1-3-Exp-F, similarly, the protective bases CTA (italicized) and restriction endonuclease *Nhe*I (underlined) were added after the downstream primers of ABCC1-3-Exp-R. Genes cloned into the multiple cloning site (MCS) will be expressed as EGFP-ABCC1-3 fusion proteins.

Supplementary Table 5. List of the mutant sequences used in this study

Purpose	Genes	Name	Original sequences	Mutant sequences	Position
Mutated binding sites for FTZ-F1 in dual-luciferase assays	APN1	APN1-M	TGAAAGGACAA	GTCGCGACTCA	-1059/-1049
	APN3a	APN3a-M	TGCAATGCCAT	CATGTCATGTC	-1110/-1100
	APN5	APN5-M	TAAAGTC	GCTCCGA	-1176/-1182
	APN6	APN6-M	TACAGTC	GCTCCGA	-259/-253
	ABCC1	ABCC1-M	TACAGTC	GCTCCGA	-828/-822
	ABCC2	ABCC2-M	CTGTCCTGTAA	CGCACACACGT	-88/-78
	ABCC3	ABCC3-M	AAGACATTCGA	TGTGTGCATAG	-131/-141
	ABCG1	ABCG1-M	CCCAAAGTTAC	TGTGTGTCAGT	-294/-194
Mutated binding sites for FTZ-F1 in the Y1H and EMSA	ABCC2	FBS-M	CTGTCCTGTAA	CGCACACACGT	-88/-78
	ABCC1	FBS^{P}-M	TACAGTC	GCTCCGA	-828/-822
Mimicking non-phosphorylated form	αFTZ-F1	FTZ-$F1^{T288A(-P)}$	ACG (288T)	GCC (288A)	+862/+864
		FTZ-$F1^{T361A(-P)}$	ACC (361T)	GCC (361A)	+1081/+1083
		FTZ-$F1^{T538A(-P)}$	ACC (538T)	GCC (538A)	+1612/+1614
		FTZ-$F1^{T544A(-P)}$	ACC (544T)	GCC (544A)	+1630/+1632
Mimicking phosphorylated form	αFTZ-F1	FTZ-$F1^{T288D(P)}$	ACG (288T)	GAT (288D)	+862/+864
		FTZ-$F1^{T361D(P)}$	ACC (361T)	GAT (361D)	+1081/+1083
		FTZ-$F1^{T538D(P)}$	ACC (538T)	GAT (538D)	+1612/+1614
		FTZ-$F1^{T544D(P)}$	ACC (544T)	GAT (544D)	+1630/+1632

Supplementary Table 6. List of the FTZ-F1 proteins in different insects used in the phylogenetic tree

Insect orders	Species	Name	Source	Gene ID	Size (aa)	Sequence quality
Lepidoptera	*Amyelois transitella*	AtrFTZ-F1	GenBank	XP_013189269	573	Complete
	Bombyx mori	BmαFTZ-F1	GenBank	NP_001037528	534	Complete
		BmβFTZ-F1	GenBank	XP_021206002	543	Complete
	Danaus plexippus	DplFTZ-F1	GenBank	XP_032527343	563	Complete
	Helicoverpa armigera	HaFTZ-F1	GenBank	PZC80407	642	Complete
	Papilio machaon	PmFTZ-F1	GenBank	XP_014370027	575	Complete
	Pieris rapae	PrFTZ-F1	GenBank	XP_022123650	576	Complete
	Plutella xylostella	PxyαFTZ-F1	GenBank	MZ962431	557	Complete
		PxyβFTZ-F1	GenBank	MZ962432	561	Complete
	Papilio xuthus	PxuFTZ-F1	GenBank	XP_013162169	575	Complete
	Spodoptera exigua	SeFTZ-F1	GenBank	AMP42756	561	Complete
	Trichoplusia ni	TnFTZ-F1	GenBank	XP_026727937	533	Complete
	Spodoptera litura	SlαFTZ-F1	GenBank	XP_022832324	555	Complete
		SlβFTZ-F1	GenBank	XP_022832323	563	Complete
	Manduca sexta	MsαFTZ-F1	GenBank	XP_030023965	567	Complete
		MsβFTZ-F1	GenBank	XP_030023964	575	Complete
Anoplura	*Pediculus humanus corporis*	PhcFTZ-F1	GenBank	XP_002430379	406	Partial, lacks N-terminus
Coleoptera	*Agrilus planipennis*	AplFTZ-F1	GenBank	XP_018325311	663	Complete
	Aethina tumida	AtuFTZ-F1	GenBank	XP_019866488	595	Complete
	Dendroctonus ponderosae	DpoFTZ-F1	GenBank	XP_019760698	582	Complete
	Leptinotarsa decemlineata	LdαFTZ-F1	GenBank	AJF93908	586	Complete
		LdβFTZ-F1	GenBank	AJF93909	564	Complete
	Nicrophorus vespilloides	NvFTZ-F1	GenBank	XP_017774948	603	Complete
	Onthophagus taurus	OtαFTZ-F1	GenBank	XP_022917387	617	Complete
		OtβFTZ-F1	GenBank	XP_022917389	600	Complete
	Tribolium castaneum	TcαFTZ-F1	GenBank	XP_008191374	582	Complete
		TcβFTZ-F1	GenBank	XP_015833964	564	Complete

Diptera	*Aedes aegypti*	AaαFTZ-F1	GenBank	XP_021697814	807	Complete
		AaβFTZ-F1	GenBank	XP_021697818	700	Complete
	Anopheles gambiae	AgaαFTZ-F1	GenBank	XP_556310	1099	Complete
		AgaβFTZ-F1	GenBank	XP_315680	715	Complete
	Ceratitis capitata	CcFTZ-F1	GenBank	XP_023158418	823	Complete
	Drosophila melanogaster	DmαFTZ-F1	GenBank	AAA28542	1044	Complete
		DmβFTZ-F1	GenBank	AAA28915	816	Complete
	Lucilia cuprina	LcFTZ-F1	GenBank	KNC27861	1085	Complete
	Musca domestica	MdFTZ-F1	GenBank	XP_005180647	733	Complete
	Zeugodacus cucurbitae	ZcαFTZ-F1	GenBank	XP_011190614	1169	Complete
		ZcβFTZ-F1	GenBank	XP_028899495	757	Complete
Hymenoptera	*Acromyrmex echinatior*	AeFTZ-F1	GenBank	XP_011053927	518	Patrial, lacks N-terminus
	Apis mellifera	AmFTZ-F1	GenBank	XP_006557455	823	Complete
	Bombus terrestri	BteFTZ-F1	GenBank	XP_012165635	832	Complete
	Camponotus floridanus	CfαFTZ-F1	GenBank	XP_025268214	713	Complete
		CfβFTZ-F1	GenBank	XP_025268213	797	Complete
	Harpegnathos saltator	HaslFTZ-F1	GenBank	XP_011139991	831	Complete
	Megachile rotundata	MrFTZ-F1	GenBank	XP_012138173	822	Complete
Hemiptera	*Acyrthosiphon pisum*	ApFTZ-F1	GenBank	XP_029344120	772	Complete
	Cimex lectularius	CleFTZ-F1	GenBank	XP_014252281	590	Complete
	Diuraphis noxia	DnFTZ-F1	GenBank	XP_015369051	770	Complete
	Halyomorpha halys	HhαFTZ-F1	GenBank	KAE8573197	542	Complete
		HhβFTZ-F1	GenBank	XP_014286886	575	Complete
	Myzus persicae	MpFTZ-F1	GenBank	XP_022167807	775	Complete
	Nilaparvata lugens	NlαFTZ-F1	GenBank	XP_0392866901	633	Complete
		NlβFTZ-F1	GenBank	XP_039286660	609	Complete

Supplementary Note 1. The promoter sequences upstream of the translation start site (TSS) of different midgut genes cloned from the susceptible DBM1Ac-S strain. The characters in red indicate the TSS, the putative FTZ-F1 binding sites (FBSs) predicted by bioinformatic analyses are highlighted in yellow. All the identified FBSs are upstream of the first exon of target genes.

>ALP promoter

TTGGCTGAACTGATTTTAATGAATCATGGTTAAACACAAACAAGACAAGAAAACAAACTATGATCCAAT
TCAACGCCAAACAGAAACAAACTCATAACACCCCTATTTTGTAAGATCGGAGTTTTAATATAATTAAGT
ATGTTTATAATTATCATCATTTTAGTAGTTCGATAAAACAAATCACAGTATTTCAGTAAAAAACATCAT
CCTTTACTCTAACTTAGTTCAGAGATATAAGGCGGGACTAGACGGCGCTCTCTTGGCAGTACAGAGACG
CGCGCACGCCACGTGCGGTCATTAAGCTTCATTATTCATTAACTTCATTATCATTATTTATTAATTTCA
TTATATCGGTTAATTCGCTATTTCATACGCGATGGCACTTTTCGCAGTAATCGTCCTTTTGAGCGTCAG
CCAAACGTTTGGCGCCAATTTTTTTGGTAAGTTTTCTGTTTTAGGTTTTTATTTAATTATTCCGATTTC
TTAGGTACGTAGACTCTTATCGTGTAAACGCTGAAAAAAATAAGCGTCTACCTATCGGTACGTATGGGT
TCACGAATCCTAACTAATATTATAAATGCGAAAGTAACTGTGTCTGTCTGTCTGTCTGTCTGTCTGTCT
GTTACTCTTTCACGCTAAAACTACTGAACCGATTTGAATGAAATTTGGTATACGTAAGGTCTAGACCCT
GGGAAAGAACATAGGCTACTTTTTATCCAGGAATTCCCACGGGAAAACTTTTTAATGCGAAGCGAAGCG
CGCGGGAACAGCTAGTTTTTCATAAATGTATGGCGTCGGCAATGCCGATTAAGTGGACGCACTTGTGGC
AATTTCTGTATGAAGAATACTGAGAGAGATGCGTCCGTTACGTACGATGCCGAAAGGTGGGCCTTTACA
GGTCAGTTTTTTGCAAAAATTCCCGATGAGTCGGTAAGTACTTTAAAAAAACCCACAATATAATTCGTC
ACGCTAAACTTAACACGAAAATTCATAAAAATCTTACAGACATAAGGTGCTAAGTTGAAAGTAGATACC
TACCTATAAGTTTACCAAACATTGTCTGAATCTTGCTAGAATAAGTGGTCCACCAACCCGCACTGGACC
ATCAACCCGCACTGGTCCACCAACCCGCACTAGGCCAGCGTGGTGGATTAGGCCTGAAACTACTCCTTC
ATTGGAAGGAGACCCGTGCCCCAGCAGTGGGGACTGGGTTGTGACAGTGAAATGACTATTAGTCCTCTA
CCTTTAGTCTCGCCACCCGATCTTTAGGTTGAAAATGAACACCTTTGTCTAATTATGCATGTTTTCGTA
AAATCCCAATATTTTTTGCAATAATTCTAATAACATCAAACCATGCTATGATCTACATAGAAACAGTCG
TATTTTTATGTCACTTATTACTACCATTCATCAATTTTCTGACTGTAGGTACTTTACTTACTCGTGTTC
AGTATTGTAATAATCATTAGAAAATTTCTTAAAATACAAAAAGCCGTTATATGCTATCTTGCATGCTAT
TTTCGGTTATATCTATAGTTCTATCTTTATCTTATATTCAGTGCTTCTTAATGGTAATTCTAAATAAGT
TGGTTAAAATTTCCATAGTGATGGCCCATTAGTTCAGTACTGAAAATAAATGTGGATTACCGATACTTG
TCTAAAAATAAACATTGTAATACGAAACGGGGTTACTGATCTCTGAACAAATTTATAGTTCAAGGATTT
TTTTGTTTTATATTAAAATTAAAATAAATCACCAATAAAGATTTTCAGCAAAAATCGATCCCTGTTTGG
TAATTTTAGAACTTACCAATAAGCATATTCTAATATTAGGTAAACTAACTTTAATGACATAATTATAAG
AGATAAGGGTAACTGGTAGGTAGGTAGGTACTTAAATTAATTTAATTACTTATTCGAAAAAAAACACGC
TTAATAAATAATCGGAACTAATTAATGAAACTTCAGGATCTTTAGAATTTAGCCATTGAGAACGCATGG
TCCAAGTCCGCGCTGTTCTATGGGATTTACATTTAAATGTATTGATCAAAAGCGTAGCTGATGATTTCA
GATTGCCGTCGAACGATCTGCAGCACATGCAACGATGTTAGTTCGACATTGGGATTGTCCTTTAAAAAC
TTCATTTAAACGTTTTGTTTAACAGCATTTTTAACCACAATACATATGTTAATCTTTGTCAATATCAGC
CTATCGCAGTTGCAAGTTGCGTCGTGTAGTAATTCATCAATGAGTCAATAATAATAAAACGCCTTTCCT
CGGCGTCTGCGAGTCAGTCATG

>APN1 promoter

AGCTTCCTCTCAGTGTTCACCATCACAGTCGCTGTGATCGCAAATATTCTGGTCTAAAAGGTTTAGCCT
TATAGTGTTTAGTATTTATTTCCATTTCTTTTATAAGTAGGACAGTGGACTTTACTTGTTTAATGTTAC
AAGACTGTGTTGATACGTAATTGTAAATATACCGTTAGTTTTTTGAAAACTTTACTGATGTTGTTCTCT
AATATTACCGATCGTATTTTATGATTTGTTACACCAATATTAATTATAATCATAAAATGATAATAATAA
ATATTTTTTACCGTAAATAAAATATTTTTAACAATAAATATTGTTTTTTTTTATGATGTTCATAGAATA
AGAAAATGATATAAATGACATATTTTCATCACTGCAAAACAAGTAACCTCATCACTACCTAACAAAATG
ATAACAAATTAATATATTGTAATGTCCTTGTAGATATTATGATTTTGTGTCGTCAAGCAATCAAGTCAG
CCCATTGCAGTGCAAGAAAGGGTCACTGAATGAAGAATAAACTGACACCTAGATACGTCTAGCGTACCT
GGTCAGCTACAACGCTACGCGTGCACCATAATTATCCCATTTTTATTCAATTAGAGTGACTAATCCCTT
CCCCATAGCGAAACAGCCCTAGCTAGTCTTCTAGTGTCTGCCACCGACACGATAAAAATAAGTTATTCT
TCTTTTTAGTGTTTTTGGGTAGTGCTTATGCCGTGGTGAAAGGACAACTCAGTCAGAATAGTGTCAGAC
ACTGACTGTCTAGGGTGCGCCAGATGTATATGAAGTACAGTTGATACTCTAGTTGAGGAGGCTGCTCTA
CCTCACGGCACTACGTTTACTTAAACTCTAATCCCTTAATTTTTTCAATAATCTGCTTGCAGCGTCACC
ATTAAGATGTCATAAGGAACATAAGGATTAAACTTAAAATAAAAATATCATTGAAATGATACCTATCGC
TAAAGACCAGTTCAGTAAGTACTATTAACCATGTAATAAGTTCATTTTATAACTAATAACGGGCTTTAA

TATGGAGTAACTATCATAATCCTTAATGTTCATTTTATCTCTATATCTTTGCGGTTTAAGATAATCTTA
ATAACATATTTGCCTAAAACTATGGATAAGATTACATTAATAAATAATTTCGACCGATATCTTATTATT
GTGAAATCTAGTGACTATTCAGTAGCTTTATACTGGCGAGAAAATG

>APN3a promoter

GCAGTATGGGCAGTAAGAAACTTACCCTAATCTAACAGGATTAGCTCCAATTTCGCCATAGTCGGTTAT
ATCGTAACCAGGGATTGGTTCATCAATTATACGTCATCTCCATATTAAATTCTTGACAGATGTGTCAAA
GAGTCAAAAGTCAACCAATAATCCCTGGTTATGCTCTTACCCACTATGGCGAAACTGGCATTGGCACTT
ATCCTTTCAGCATAAGAAGCCCTAGTCTTTAGCCCAGCTAGTTTTATTTGTAACACGATAAGAAGACAA
CCACCCTGACCGGAAATCAAACTTCTACAGCAGCTGTCTTCAAACACTGTGCCGTCAAAACAGGAAAAA
AACTGGGCTAGACCCAAAAAGTGCTCGTTAACAAAACAAAGATTACGTATCGCAAGCATCATTCATCCA
GGATAGATCTAGATATGATTATGTTTATCAATCGCTATCGGATTATAATAATTTGTACCTTTCAAATTT
AATTCCACCTATAGTTAGTGTCTCTAATTTGATAAGGCATTTAGTGTGTTGCTGTCGCCAAAATAACAA
GGTAAGTTGGTGTTATTTTTATTTTTCAAAATAAAATATAGAACCACCTAACATCTTACTAACAGTATA
GAAAGCATACTTCTGATTTTCATTAAAGTTTCATTTTCATAACTCGTAGAAATTGATTACTTTATATTT
TATTATTAATTAAATGTGAACTCCGCCTTATAATTATGATAATCTTACATGGGCACTTATTTACACTCT
TGGACAATGAAATACCTACTTACTGTTTTTTTCCTAGTCTAAACCTGGACACTGTTGACTGATGGAAGT
TTTTTGTTTCTGTCTGATTCAACCAACTCAGGATTAATCAGTATTAGGACTACAATTTAAAATTGCGTA
TATAATAAAGGCAACCGCCAAACCTACAAGTTTGCAAAAACGATTTTCATAAGAGATTATGATTTTTTA
TCGATGAGAAAGCATGCTTAATCTAATACAATGCGGTTCCTTAATAGGCGGAATAGGGCGCTTAGCTAC
TTCTTATGCCTACTTTTTATACTAAGGCTGCGTTCCTAGTAAGCAATAACTATTCGCCACCGCGAGTGC
CACGTAATTTGCAGCGACATGCAATGCCATTCATAAGCACCGTCGCTCGTCTATCAAAATCTTCCAGAT
CCACACAAAATAAGTCAGATTTGACAAGCTTCGACGCAGCTTAACGATTGCTAATGTTCGGTGGAGGTA
ATCCCACAGTTACCCTACTCGGAAAAACCCAAAAGTTTGAATGTTTTTGTTAATTTAAACAGAAATGTG
TTCTGTAATCCTTAACCATCTAATTTGATTTTAAAACTTTATTATAGTCTTTAATTATTATACAGGGTG
ATGCAAAAAGGGTATAGGTACTAAGCCGCAACCTACATGTGCAGCATGTTATAATAATATGTAAGCTAC
AAAGATATTTTTTTTTCCGAATTTAGAAATTCTGATTTCATTTTCGGGCTTAGATATACCATGCAGCAC
ACGTAGGTTTCGGCTCAGTATTAGTATACCCTTTTTGCAACACCCTGTATAAATAACTAATAGTAAAAT
GCCTGAACAGATGCATATCAAAGGTATCTAATCAATATATCCGGTAGGTATATATCAATTTTTAACAAG
TAAAATACCCATTTTATTGATATGACTCAATAAAATAATAATTATTTTAATTATGTTATTAGGAATAAA
GTGGTAGGTAGATATCAATGATAACATCTTATCTATCTTTAAGCATATTATAACTCTTTTAACAATATC
AAGAGATAATAGCTCTGCTCTATCAGTCAGTTTCTTATGAGGTTTTTGTGTCCCATGCTATAAGGATGC
TTTGTATGTAATGAGTAATGACAGTTGGACAGCGATCTGTATCTCAGCGGGTCTGGAGTACTTTTCCCT
ACCAAAAACCTTTTTTGCTCGTCATTTTATTGGCGGAACCCCAGTATAGGCGGACAGCCTATTGCAATA
ATACATAACATACAAATTTCTTTGCTCATTATGTTTATCAGACCTGTACCTGCAGTTTGGAAGCATTTT
TTTTTTCATAAATTGCGAGGCTAACCAGTGTATACTTACTTCAGTATTCGTTATTTGAACACAAAACAG
GTTGATAAAGTCTTTTTCAGCCTTTCGTTATTATGTTTATTAAATCTGTCATCTTTGAACAAAATTTTT
TCATTACTTATCTAATTTATTTTAGATG

>APN5 promoter

CGCCACGAAGGATGGAGCAGCACAAATTGTTGCTTCAGCTACTTTGATGATCTTATTAGCAATGATTAC
TTTGATAGTTTAGAGTTACGTTGACATTGCCGTCGATCAATAAAACACAAAGATTTGAATAATCTAGGT
ACTGTTGTTTCATTGATTGCCATTTGAGATTACACGAATAATATATAACTAAAAAACGTACCCTACATA
GGATATAAATTTAAAAAAAATGTGAGAGTAACAGTTATTAGATAGTTGGATTAAATAATATTTTATCGA
TACGAGTACATACCTACTTACATTCAATTTATACTGTTGAATTATTTTATATTTTACCTATTATCTAAC
TAGGCAACTAGGCTACTGGTTATAGTAGGCATGACCAAATTAAGCACTTTGATAATAACTCCTAGCAAT
CTGAACCACCCAGCTACAGTCGGTTTCCTATCATAATATTTCATCTAGCATCTTTCATAGCATGCATAA
CTGCATTTCCCACCTAGCCTTAGGGTATACTGGTTGAGCTAATATTGACCTTCCCTACCCTAATATTAG
CCTTATTTATTTTATAGTATTAAGTGGAGGTTAAGGAACCCAGTAAAAGTAGTATTTTGTAATGTAATG
AGAGATTTTGGAAAACCCTAAAAAACGCTTTTTATCCCATAAACATTTCTAACGGATCAGAGCGAAGG
TAAGGATTGTATTGGAGAAAGATAGAGAAGAGTGGAGGGAATTCTATTCTATTCTATTCTATTCTCTGT
GGGGGTGTAAGTACCTGCACCTGGCTCTCTCGAATGGAACCGTTGTGAATATCCCCAAGGTCTAAACTG
CCTTCCTAAGCTTGGACCATTTCCCACCACGCTGGTCCATTGCGGGTTGGTGGGTTCACATATCTAGAT
GTGCTAGATCTAGATATGCAGGTTTCCTCACGATGTTTTCCTTCACCGTAAGAGCGATGGTATACATTG
TACTTAAGTTAAAAGAACTCATTGGTACATGTCAGCGCTGGGATTCGAACCCGCATCTCTGGCGTGAGA
AGCGGGCGCTTACCCGACTGAGCTACCACCGCTCCCGACTGAGCTACCACCGCTCCGAGTGGAGGGAAA
GGGAGGGAAGTATTTACGGAAAATCCTTTAAGGAGCAGTTAAAATTGCGGGCAGCAACTAGATACTTTC
TTATAAAAAAAAAAAAAATTGACTTAGTCGGGAAAGATCTGTTAAATCGCTTATCTTTGCAGTTGTAAAT
GAATGAAACAAATTGTATTTATTATTATTTACAGAACTATGATAGATGTATCTTATCGATCTGATAAAT
TGTAATGCGCCGATATACATAATCTAAAATACGTAATTATATTTAGATAAAAAAGATAAATTTAAGAA
CTTCTGGCTGTTGTTAAAATAATTTAGTCATTTATTAAAAAGGATAGAGCCACAAAGGTAGTGTTATTA

AAATTTTACATTTAAAGCTAAGCAAAGTGCGTAATAATAATATGGTCAAACATTGACTATATTTTTTT
ACTTAATTTCATTAATTTCAGTAATAAATTATAATTCTACGATATTTTTGGTGGTGCTTAGTAATAGCG
AAATAATATTTTCTTGTAGAAGCATG

>APN6 promoter

ATCGTCATTGTTTGCTCCTGTTACTATGGTATTAGCTGCTGTAATCCTACATTTGTTTTACTGAACTAT
GTTTTGTTGTATTTAAGATTTGATTGTAAATAAATGATGTTTTGTTTGTAAATGTAATGTTTCATTAAT
TGCATTATTCTTATTTGAAGGATAGTTTGATGTAAAATTCCATGTAACATTATGAAAAAATTAAACTGC
CTTAGTACCTAAGTACCTATTTACTGTTTACGTAATAATCTCTTTATCAGTATCATACTACTAAATAAT
AAGGGCAATTACCATACAGTCTTAATCGGCACTAATATCGATAACTATAGAGATAAGATAGGTAACTAA
ATAATTTGATAACTTAAATCCATTTTATTAGGTAGGTTTTTTACTTGTACAGGGCAAACCTGCTGATCA
GGTAAGTACGTTGATTTTATTTTACATTTGTTTACAATTACAAAATACACACAATCTCTGACAAAATTG
AAATATTTTAAACCATATCATAAAGTGTCTTGTAAGTGTATAAAATAAATTTAAAATATTACAGGCATG

>ABCB1 promoter

TTAGTTACATAGTTACCGACACACAGCTCTATTTCACCCATGGGTTGTCGTTTTTCACGATAAGGCTAC
TTCTTTATGTACTTACTACTAATTATCCTGTTTAAGTGAACTAATCTTATGATACCTACTTACCCATCC
CATGATTCCCATGAGACCATGACTACAGCATACTTGCATAACTAATGGCAACCGCTTAACCGCTACTAT
TCAAATATTCTTGCTGATCGTGATGAAGCCATGAAGTGATGATTGATGACATACAAGGTAACGTTAGTT
TATTTACTTCTATGGTAAGACCCACTGCTATTACTTACCAAATTCAGGAGAGTTCACTATCTATTTGTT
TTACCTAGATGTTACGAGAACTAGGTACGGTCAGGTGCAGAAAAATTTGACCCCCCTCTCATAGTAACA
ATGATTCTGATGGCCCCACTACACGTCCGTGGATGCGTCCGAAGACGTACGCGGATGCATCTGCAGACG
AATCTCTGCAAGCGACCACACGTCTGTTGCTCGGTCTGTCTGAGTTGAATATCGGCAGACGCTACTCGT
AAACATGGACATCGAAATAGCAGCCGCAGCAGCTATTTATGTGTTTAGTACTTTTGACTACTACTTCAG
TTTGCAGTCGAGTTATCAAGAAGAAACGGAGAAGAAGACGATTCTGGTCTCACTGTCTGAGGCGTCCGC
GGACGTGTGGTCACGAAGCCGGACGCTCGCGCTTGAACCCTTTGATCTGTGCCAGAAAAACCGACAACA
AGCGGATCGGTGACAGATGCGTCTGCGGACGTGAAATACCTCGACAGATGCCCAAATCACCCCCTCTAC
ACGTCCGCGGAGGTCATCTCCGGACGTATCCGCGGACGTGTAGTGGGTCCGGGGGGGGTCAGGTTTCTC
TGCCCCTTACTGTACTTACTTAGATAAGTAAGTAGGTAATTATAGAACTTAGCTAGGTAGGTCCTAGTT
CTACCTAAGTATATCTCATTTGCCTGTAGCATGTCTATCTAAAACTAGAAAGAAATGAGAGATACGATA
TCGATGATAATAATAAATGATAACTGGAGTTGGTATTTGTGAGCTTATCTTCCAGGTATTGATAATAAA
TTACAATAAGGTTTAGTCAGTGGCAGTTTGCGGAACACACGGGGATACTCCAACTCATAATGTTTATAA
TGTGACGAAAACTAAATTGTTTATTTAATGATAAAGATATAAAAAACTAAAAGATATAAAAACGTACAG
TCAAAATG

>ABCC1 promoter

CGTAGTGTCTTTCCCTCTGTAGCCGAACCAGCGTCAGCTTTTCAAGCGCTGAATGAAAACCTCAGCACT
TCAGCGTCACTTCAGCGCGTGGAGTTAGGCAGTACCTACCTACATATTAATAAAGTGTATTTGCATCAG
CACTAGAAGCCAGTGTTCTACGTGTATACGCCCTAACACCGCCACTAGACTTGTCGATAAACTGTTATT
GTGTGGATGTATTGTGACAATTGTAGCCGCCGCACAGACTACAGGCGTCTACTCGATGACCCCGCCACT
TCCCCGCCGCCGCCGATCGCCGAACACAACAATACCGATCTGATTATTGAGATCACATGTCCACGGAAC
GTGGCTTAATTTACATCGGATTAACCAAGTTAACATGAAGCCAGATGATGAACGGTTTTGCATGTGTAT
AGGATGTAATTTTTTGTGTCCACAATCAATTTTCCATACCGGAAATGCACAAAGCACATTTTTGAGTCA
TTTTGAGTATTTATGTTTTTTTCGTTCAGTTTATCCAGGAGATCTCTTGGATTAACTTAATTTGTCGAG
CTTTTGCACTCTGCATATTTTAGTAATCTCTCTCCCAATTTGTATAATTAATTAATGCTTAAACTTGTG
TAAGTATAAACTTCCAACAACATCTAATTTATGAAGCCAAAGTTTAAGTTTAAAATGCGCCATTCATTC
TCATTGCAGCGACATTCGAGATCGAAAAAATATAGAAAAACAAGTCTAAGTACCTATGTACTGTCGTGT
ACTCGTCTACTCTTGATGAGTCACATGCAGTTTCGCTTTAGCCAGATCTTATATGTATTAAGCGCCAGT
TCTACAAGTTGCGGACCAGTTCTACATAATCTGTGGACCAGTTGGAGAAAAGGCTCGCTAAGATAATTT
CTGTAATTAAAAATAAAAATAAGCAAAAGCGCATGATTAGGTGGCCACTCGACAGTTGCCAGGTCTTGA
TGATTGAGCTTTGATGGGTGTGAACGCAGTACAGTCACGGATAGCTAAGAGGATATTTCAGGTACCTAC
TTACTTATACAATATTTATCGATATGTTTTAGAAAAGTTACAAAGGTTAGATGAGATCTTTTTCAAGTT
TTTTGTAAATAATCTGCCATTATCAATTATGATGTACATTATTACTACAAATCTATCACGTAAGACAGT
TTGAATAAGTTTACTTTAAATATTTTTGTTCATTTTAAATATATTTATTCATACACTCCAATAAAAAAA
TATTCGCTTAATTTGCAGTAGTCCCATCTTTACATCCTGGTTTCTTCAAGCTCTCTGATGTAGACCGAG
CGGACAGGACAGTGAGGATAAGTTGACAGCCTAGAAGCGGTAACGGGGCGACGACGACACCTATAGATC
TATACAAACACAGCGGCGCGGCCGGCGCATGGCACTGCCAGGCACACTCGTTCCATATGCTTAATAGGT
TTATTTCTGACTGCCAAGTGAATAAACAGCTTACAAGTAAAAAGCTGCATTGATTCCTGGTATTAGAAA
AGTAGATACACTTAGTACATAATCAAAAAGTATGTGGAGGGACGGAGCGGTGGCGCCGCCGCGCCGCTC
GGCTTCATATAAACTGGCGGGCGGGGTGCGCGCGCCAGCCAGTAGGACGCAGGTGAGCGCTCGCATCGT
TTGTTCGCAAGCGCACGCGCACGCGGCCCCGCGCCCCCCGCCGCCCCCCGCGCTGCCCCGCACCATGCT

GGTTCGCGCTCCGCACCCACACTCCTGATACAGTGACGATAGACGTGGACTAAAGCTTCAGTTTTATAT
TTAGTTGTAGTTACAAAAACACATTCAAAATG

>ABCC2 promoter

ATTTCCCGATAGGCTGTGAACTATCTGAGGTTGCTTCTCAGAATTGTGATTGTAAAACTGTTCCAATAT
GCCACGTCAAGATGTTAGTCGAAGATATTGAAAATAGATTAGCAGACTGTAAATGTGTTTGTGACTCTC
TGATTCCTAAAGTATGCCCAGTACATTCTTTCTGCAACTAAATTACTGTAATTAGTGTATTTTATAATT
ATATGTATTTGATTATTTTGACAATATGCTTTTTCATTGATATTTATGTAATTTTAGGGACGTTTTCGG
TGTGTTATTTAATTACGTAAGTTATCGTTAAAATATGTGCGATAGTAAAAATATGAAACCTTAATTAAA
ATCGTAACCGTATTATTATATTAAAAGCTTGTTTCATTGTACCTATACTTAGATAGTACTTATAGTAAG
TGCCAGGTATTCTAGGTAACGGAGGTCTAGATATACATTTGAAACTCAAAATGCCAAGTTGAACGAAGG
AACTTTAACTAACATCGACCTTATCGAGCGTTTGTAAGGGCCAACCGCTCTAGAAAATTTCACCCTCGC
GTTGGCCCTAAAATCTCATAAATCTGTAATAATTTGAATGAATTAGGGCCAACGCGCGGGTGTCATTTT
CTCAGTGTGATTTTGCAAAGTATTTTTATCATTGTCAGTTATTTACTTATAGTTAAATATATTTTATAA
AAAACCGATCATACTATATTTTTAACGAATACCTATGTCCTTGTCCCTGCTGAGGGACGTCAGGATGGA
TGTATGTATGTATGTATGTCCTTAAATAAAAGTAATACGGTCTTTAAAAAATAATTAGGATCAGCTGTC
TTGCAGTGGCGCACGTTAGTGGAGACCTTCTGCACCTCTGGTGTGCCATAGGACTGCAACAACAACAGC
TGTCGTGCAATCGGCACCGTCGTATTGATATACAACGAGATAAGTCGTGGCTCACGAAGCAGCGCTCCG
AATTTTTCGTGATATAGACCCTCCAGTCGTATTTGACGACCGAATCCCGAAACCCTGTATAAGGATTAT
TCTATTTTGGTATTTTCTATTTTGCGGGACGCCACAAGGCATGCATCGTCCCATAAACTAAAACGAAAA
TGAACACGAAGGTTTCGTGCGGCCGCACTGAAACATATTACGCTCATCTTGTTTGTATCGATGATAATG
ACTGTGTAATAAATTGTTACCGATAACAAATCAAGCGCCACTACTTAACAGGAGCGATTATTAAATATT
GACTGTTGAGTGAGACGGACGTTTGTTGTGCAACCTGTCCTGTAAAGTGTTTCTTGGAGATACCATTGT
ATGGGTATTAGGTGTGTAGGCGCAGTTCAGTGGTGCAAACTACATGTGTCGAAATG

>ABCC3 promoter

AATACCTCACCTGCCCACCTACTTCCACAGGTTAAACGAAACCCATAATCGTGCCTTATTAGCCGAAA
ATCGACCCCTACCGCATGGAAATAAGTGGGGTGGAGGGCCGCTCGGTATTACTCTGGCGCTATAAATC
ATATTTCGCTTTTGGCATTGATGGAAAACTGAATTGTACTGCTCTGTTTGTTATTTGCACCAGAATTA
TAAATAATATTTTAATGATATTATTATTTACTCTCTGCGGAAGTTAAAGGACATGGGTTCAGATCAGG
CATGTACCAACGATTTTTTTCAATAAAACATCGTCGAGGGTTTCTTCACTTTTAAATTTATGCGAAA
GATGAGGGTGGGGGACAGGCTAGGTTTCGGCTTAGTATAACATTTATGCAACATCCTGTATATGGTCC
CTTACCATGTTTACGGAACCCTTCGTAAGTCAGACTGACGCTTGGATGGTTTTTAAAATTGTTTATAC
TCGTATCTACACGCAATCCGATAACGATAGAAAGCCCGATGAACCGACCACAAAGTTATCCTATAAGA
GTCCCGGTTTACCTTTAACGGTACCTATGGATCCCTTAACTTAAAACCTATGTACATGGAAACACTTT
AAATTCGATATGTAAAACGTGGATTTAATTCTTACATAATGTTCTTGCTCAAGCATGACACCACTTCC
TTTCAGTGATACATTAATTAAATACCAAGTAGTTTCTTTAAATCGAAGACATGAAATAATAACCAAAT
TAACTATAAAACGTGTGTGATTACAGGGTGTTGCAAAACTGGTGTATTTAGCCGAAAGGACCAAGTCG
GCTGGTTATTCTAAACAACTTTTCAAAATTTATGAAAAATAACTACTCCTCATTGTAAAAAATAACGG
AACCGTCTAGTTCCTATTATGTGCAATATTTTTTTCGCAAATTTTCAAAAGTTGTTTAGAATGACCCC
ATGAGTCACATCATTTCGGCTTACTATATAATATGTACATATTATACCCATTTAATACTCATTCAAAA
CATTAATTGACTGCCTATGAAATCTGATTTTGGTGTGATTTAATTAACTGTCGTACTAATTTTGCCAT
TCCCATATTATGTATTACTGTTGCGCCTACATTTTATTCTACTTATCGCTGTAGTGCCCGAGAACTGT
AAAAAACATCATGAAAGTTCTATAAAAATGAGCAAATACGGTATGAGATACGAGTGGTAGATGATAAT
ACACAGTTATATAAGTAAGTACTTAAGTAGATAATATGCATATTCATTATTCGTCGAAGAGGTCTTTC
GCTTTACGACCATTCCTTTAAACCCAGTTATAAAATACCTATAATAATAAATAATGCTGTGTTCCTTG
TTTAAGTTTTCTATGGATCAGGATTAATTAAAAAAATATTCTCACGGCTCTACTTCCTAAGTAGGAGC
TTTTACATCACACAACTTACACTAATAAAATAATGCAAACATACTGTATGTGTATATGCGTACGGATA
TTTATTTCAATATCTATATAGTACGAACTCCTCTGCTTGTAGGTAAATAATTCTTTATTGCACACAAA
CATAAAAATTACAAAAAGAACTACACAAAATAGACGGTACAATTCGCGACCTTATTACTAAGAGTAAT
CTACCAGACTACCTCGGAGGAAAGAAATTATATGCAAGATAATGTGAAAGTACAAAAAATGATATATA
CCTACATTAATAACGACCTCCCACTTATAAATATTCATATTTAAGGGCTGCAGTTGTAACTACAAAAT
AAATAAAAATGTAGGTAATGAGATACAGTAGTTACTTATACATATCGTATTTTTTGCAAAGCGAAGAA
ATGGTCCGAACGAAAACGCGTCGCTTCTCGCCCTAGAGACGGCACGTAATTCAGGATAAGATTATAAT
GAATATTTGATATCAGATAGGTCGATGCGGATCGTCATACACATAAACGGTGTAACTACTTAATTCGC
GCCGTTTGATAAGACATTCGAGTGTGGCGCGGGCACCAGCGAACATGTGGCTTGTGTTTGTCGTGCAT
CAGTTGTTTGTTTAGTTTTTGTAAATAAAACATAGGTTTATCTTAGTAATCTAGATAATTAATTAATT
AATAGTGATATAAGAATG

>ABCG1 promoter

ATGTCGATGTCGGTGGTTTAGCCGCCGACCCACTAATTAAATCTAGATATTTTATTCACTAGAAGTCTT

TATATCAAAAAGCGATTTCTAATCATTTGTACCTATTGAATTCTCGTATCGTTTATTTTAATGTGAATA
AAAATACCAGACATATCTAATTACGACATTCATAAAACTTGTCCAATTAGATTGTATCTTGCTTTGAGG
CTGATGACATTTCCATTCCTCGCCGTACTATACTATTTTATTTCCTCGAATAGAATACGCTACATTTTA
TATATTTTAAAATGGTTAAAATGCATTAAACTGTCTTATCGTATCTTAGGCATCAGTTTAAAATCCTAT
TTAATTCTTCTGTTTAAAGTAAGCGTCACTACGGTACATTTTTATTTGCATAC**GTAGCATTGAA**CAAAT
CCATAAACACTGCCAATTATTGAACCATAGACTGAACGGCCGCGCATTGTGATTGAAACTAAATGAAGA
CAAATACAGTTCCACCATTATAATAATTATGAAACAGTCTACTTACAGATGTAATTTAGCAAAATAACC
AGATTTAACTTTACCGATGTTCCATTATATCACAATAAATAATAGTGAGAACTTTAGAAATAAATCCAT
AGTGTTGTAGGTACTCACTTTATGTAGGAATTAAATTTTTTGTTCCCTTATCAAGTAGGTATGGTAAAG
CAAATATTTAAGTTCAATATAGGTCCAATATGATCCATTTATTTGATATAACATTAACTAACATAACTA
GTTTGAATATCTTCTACATTTATTTGCTAATAAAAAAGCCACACTGTGTAAGGAGGTATAATTAAGTAA
TGCTAATTAAAATAAGCGAGTCATACAACATTTCATAATAGCTCAAAGGTATTTTAAAATACCATTACT
GGTCATAAAAAATAAAGATTTGCTATTTTTTTTTTTGCAAAGCATTCAATCGCTACAATATAAACCTTT
TGGACAAAACACATGTAGGTATCATCAAATCAATAAATTATATAGCCAAAATATATGATAAACTACATT
AGTATGTACCTATGCTAATAAATTAAACCATTAATTTAGGTTAATACTGTTATAAGGACTATTAAATTC
ACGAAAAGCTTCCTATGTTTGTGCTAAATAACTAATATTACTTCTAGTTGTTAACCATCGTTAAAAAAG
ATTATCTTTTTGTGCAATGTAAATAGATACCATTATCGTATTTAATATTATACTTTCTTACCTAAACGA
ATAAAATAAAATAAAAAATATGTTCATGGGAATG**GTCGAGGTTAA**TTCGCTTATGTAAGTAACCGATTG
AATAAACGAAACGTATACCTAAATACGCTTTGCTACTGATGTTTAATGTGTAATTAAATTATTGTTTTA
TTAGATTAACATTATGAATGAAACGGTCTTAGTTAAAACGCTTCGATTGCGTGTAAGTACGTCGAACTA
CGGACGCTTGGTTAGGCGGCAATAACATAAAATAACTAAGAAAACATACTTAGTAAGTACCTAGGAATA
GGAACAGGTCTATAACGCCTGGCCGAATAGAAGATAATTCACCAAATAATAAATATAAATTAAATACGA
ATCAAGGTTTGTTGTATGTTATTCGCAATGTTATTGTCAAATAGAGCATAAGTTTACATTTATAATAGT
TATGATAGCCATATTGCTAAATCATATTTAAATGTAGGGTTCATACATCATCTCAAGTCTGCATACAAA
TAGTTCCTTTGTCATGTGTCATTTTTAATCGTACTCGTATCGAAATGTAAAAGGTATTTCATTATAATT
AAAATATTGTATAAATAAATAATTACCGAATAATTATTTGCTAAGCTCAAATATGTTTTTTTCATAAAT
AAACATTTACATACGATTATTTTTGTATTAATTTCTAATGTATAAACCTCCTAAACATTATCAAACTTA
ACAATCCTTGTACTGTTATTTAACTCACAGGCACGTCCTTTGTGTATTATTAATAACTGTCTCTTTGTA
ACAGCTTATCTCAATCCCGAACTTCAATAGACATGAAACTACATTTCGCGCTAAGCAGAGCCAAGCTCT
GCTAGATGAAACACCTC**CCCAAAGTTAC**CAGTCCAGAGCTCATTAGAAGAGACACGAAGAGTAAGGTGC
CGCTCGAGTCAGTAGGCTCATTTCGACACAACGTGCGCGCGCGGCCGACGCAC**GATACCAAAGACAAA**C
CCGCCAAAACGAGAAAAAAATAACTTATTGAGTGCCAGTGTGTGTTGTGTGCGTGATTGCGATTAGTGT
TTTTTTTTTACAAA**ATG**
