# Supplementary material for: A single transcription factor facilitates an insect host combating Bacillus thuringiensis infection while maintaining fitness
Source: Nat Commun. 2022 Oct 12;13:6024. doi: 10.1038/s41467-022-33706-x (PMC9555685; doi:10.1038/s41467-022-33706-x)
Supplement: Supplementary file 2 — Description of Additional Supplementary Files [file 41467_2022_33706_MOESM2_ESM.pdf]

Title: Supplementary Data 1.

Description: Identification of the phosphorylation sites in the recombinant FTZ-F1 protein of *P. xylostella*. (excel file)
