# Supplementary material for: A single transcription factor facilitates an insect host combating Bacillus thuringiensis infection while maintaining fitness
Source: Nat Commun. 2022 Oct 12;13:6024. doi: 10.1038/s41467-022-33706-x (PMC9555685; doi:10.1038/s41467-022-33706-x)
Supplement: Supplementary file 4 — Reporting Summary [file 41467_2022_33706_MOESM4_ESM.pdf]

nature portfolio

Corresponding author(s): Zhaojiang Guo, Youjun Zhang

Last updated by author(s): 15/9/22

Reporting Summary

Nature Portfolio wishes to improve the reproducibility of the work that we publish. This form provides structure for consistency and transparency in reporting. For further information on Nature Portfolio policies, see our Editorial Policies and the Editorial Policy Checklist.

Statistics

For all statistical analyses, confirm that the following items are present in the figure legend, table legend, main text, or Methods section.

n/a	Confirmed	
☐	☒	The exact sample size (*n*) for each experimental group/condition, given as a discrete number and unit of measurement
☐	☒	A statement on whether measurements were taken from distinct samples or whether the same sample was measured repeatedly
☐	☒	The statistical test(s) used AND whether they are one- or two-sided<br>*Only common tests should be described solely by name; describe more complex techniques in the Methods section.*
☒	☐	A description of all covariates tested
☒	☐	A description of any assumptions or corrections, such as tests of normality and adjustment for multiple comparisons
☐	☒	A full description of the statistical parameters including central tendency (e.g. means) or other basic estimates (e.g. regression coefficient) AND variation (e.g. standard deviation) or associated estimates of uncertainty (e.g. confidence intervals)
☐	☒	For null hypothesis testing, the test statistic (e.g. *F*, *t*, *r*) with confidence intervals, effect sizes, degrees of freedom and *P* value noted<br>*Give P values as exact values whenever suitable.*
☒	☐	For Bayesian analysis, information on the choice of priors and Markov chain Monte Carlo settings
☒	☐	For hierarchical and complex designs, identification of the appropriate level for tests and full reporting of outcomes
☒	☐	Estimates of effect sizes (e.g. Cohen's *d*, Pearson's *r*), indicating how they were calculated

Our web collection on statistics for biologists contains articles on many of the points above.

Software and code

Policy information about availability of computer code

Data collection	Inbuilt software associated with the following equipments:<br>Dual-luciferase assay: GloMax 96 Microplate Luminometer; qPCR: QuantStudio 3 Real-Time PCR System; EMSA and western blot: Tanon-5200 Chemiluminescent Imaging System; confocal: Carl Zeiss LSM 700 confocal microscope equipped with ZEN 2012 software and Leica TCS SP8 confocal microscope; LC-MS/MS: Orbitrap Fusion Lumos Tribrid mass spectrometer coupled to an EASY-nLC 1200 system
Data analysis	Gene sequence analysis: DNAMAN 9.0; multiple sequence alignment: Clustal Omega, GeneDoc 2.7; TF binding site analysis: JASPAR, PROMO, WebLogo 3; gene primer design: Primer Premier 5.0; protein sequence analysis: Translate, NCBI's CDD; phylogenetic analysis: MEGA 7.0; western blot: ImageJ 1.51; LC-MS/MS: Proteome Discoverer 2.4 and Disorder Enhanced Phosphorylation Predictor (DEPP); statistic analysis: SPSS 23.0; figure drawing: Microsoft Office 2010, SigmaPlot 12.5, GraphPad Prism 8.3

For manuscripts utilizing custom algorithms or software that are central to the research but not yet described in published literature, software must be made available to editors and reviewers. We strongly encourage code deposition in a community repository (e.g. GitHub). See the Nature Portfolio guidelines for submitting code & software for further information.

Data

Policy information about availability of data

All manuscripts must include a data availability statement. This statement should provide the following information, where applicable:

- Accession codes, unique identifiers, or web links for publicly available datasets
- A description of any restrictions on data availability
- For clinical datasets or third party data, please ensure that the statement adheres to our policy

The full-length cDNA sequences of all the cloned genes in this study have been deposited in the GenBank database under accession numbers MZ962431 (https://www.ncbi.nlm.nih.gov/nuccore/MZ962431.1/) and MZ962432 (https://www.ncbi.nlm.nih.gov/nuccore/MZ962432.1/). The gene or genome databases including DBM-DB (http://116.62.11.144/DBM/), LepBase (http://ensembl.lepbase.org/Plutella_xylostella_pacbiov1/) and GenBank (https://www.ncbi.nlm.nih.gov/) were used to obtain sequences of target genes and their promoters. The authors declare that the data supporting the findings of this study are available within the paper and its Supplementary Information. The source data underlying Figs. 1, 2, 3a,c,d&g,h, 4a&b, 5, 6, 7c-f and Supplementary Figs. 2, 5, 6, 9b and 10b are provided as a Source Data file.

Human research participants

Policy information about studies involving human research participants and Sex and Gender in Research.

Reporting on sex and gender	n/a
Population characteristics	n/a
Recruitment	n/a
Ethics oversight	n/a

Note that full information on the approval of the study protocol must also be provided in the manuscript.

Field-specific reporting

Please select the one below that is the best fit for your research. If you are not sure, read the appropriate sections before making your selection.

☒ Life sciences ☐ Behavioural & social sciences ☐ Ecological, evolutionary & environmental sciences

For a reference copy of the document with all sections, see nature.com/documents/nr-reporting-summary-flat.pdf

Life sciences study design

All studies must disclose on these points even when the disclosure is negative.

Sample size	Sample-size calculations were not required for all experiments of this study, sample sizes were selected based on our previous experience to obtain statistical significance and reproducibility [Guo et al., 2015, PLoS Genetics, 11(4): e1005124; Guo et al., 2020, Nature Communications, 11(1): 3003; Guo et al., 2021, PLoS Pathogens, 17(9): e1009917; Sun et al., 2022, BMC Biology, 2022, 20(1): 33; Guo et al., 2022, PLoS Genetics, 18(2): e1010037]. All samples sizes are indicated in figure legends or the corresponding methods.
Data exclusions	No data were excluded from the analyses.
Replication	Experimental findings were reliably reproduced in at least three independent experiments as indicated throughout the manuscript.
Randomization	All insect individuals in our experiments were randomly allocated into different experimental groups.
Blinding	No blinding was undertaken. The work presented here involved empirical measurements and was not subject to subjective bias. All experiments involved independently performed biological repeats.

Reporting for specific materials, systems and methods

We require information from authors about some types of materials, experimental systems and methods used in many studies. Here, indicate whether each material, system or method listed is relevant to your study. If you are not sure if a list item applies to your research, read the appropriate section before selecting a response.

Materials & experimental systems

n/a	Involved in the study
☐	☒ Antibodies
☐	☒ Eukaryotic cell lines
☒	☐ Palaeontology and archaeology
☐	☒ Animals and other organisms
☒	☐ Clinical data
☒	☐ Dual use research of concern

Methods

n/a	Involved in the study
☒	☐ ChIP-seq
☒	☐ Flow cytometry
☒	☐ MRI-based neuroimaging

Antibodies

Antibodies used

All the primary and secondary antibodies used in this study have been described in detail in the manuscript.
Anti-FTZ-F1 (produced in this study, 1:10000); anti-Cry1Ac (produced in our previous study, 1:100); anti-rabbit IgG (CWBIO, #CW0103S, 1:5000); anti-GFP (Abcam, #ab6556, 1:1000); anti-mouse IgG (Sigma Aldrich, #12-371, 4 μg); anti-Alexa Fluor 555 (Abcam, #ab150162, 1:500); anti-β-actin (Abcam, #ab8227, 1:2000); anti-Histone 3 (ABclonal, #A2348, 1:2000).

Validation

The validation of anti-FTZ-F1 and anti-Cry1Ac prepared in our laboratory has been respectively provided in this study and in our previous study [Guo et al., 2015, PLoS Genetics, 11(4): e1005124].
The validation statements of commercial antibodies are available on the respective manufacturer's website.
Anti-rabbit IgG (Species: independent; application: WB, ELISA, IHC); anti-GFP (Species: independent; application: IHC-P, Electron Microscopy, ICC, IP, Flow Cyt, IHC-Fr, WB); anti-mouse IgG (Species: not confirmed experimentally; application: IP, WB); anti-Alexa Fluor 555 (Species: not confirmed experimentally; application: IHC-Fr, ICC/IF, ELISA, IHC-P, Flow Cyt); anti-β-actin (species: not confirmed experimentally; application: IHC-Fr, IP, WB, ICC, Flow Cyt, IHC-FrFl, IHC-P, IHC-P, ICC/IF, ELISA); anti-Histone 3 (Human, Mouse, Rat; application: WB, IHC, IP, ChIP).

Eukaryotic cell lines

Policy information about cell lines and Sex and Gender in Research

Cell line source(s)

The insect cell line Sf9 was purchased from Invitrogen, the insect cell line S2 was donated by the Institute of Zoology, Chinese Academy of Sciences.

Authentication

The insect cell lines Sf9 and S2 were obtained from original source and were not further authenticated.

Mycoplasma contamination

The insect cell lines Sf9 and S2 were tested negative for mycoplasma contamination.

Commonly misidentified lines
(See ICLAC register)

No commonly misidentified cell line was used.

Animals and other research organisms

Policy information about studies involving animals; ARRIVE guidelines recommended for reporting animal research, and Sex and Gender in Research

Laboratory animals

Diamondback moth, Plutella xylostella.
The five different P. xylostella strains used in this study have been described in detail in the Methods section of the manuscript and our previous studies [Guo et al., 2015, PLoS Genetics, 11(4): e1005124; Guo et al., 2015, Journal of Invertebrate Pathology, 126, 21–30; Zhu et al., 2015, Pest Management Science, 71(2): 225–233; Zhu et al., 2016, Pest Management Science, 72(2): 289–297]. The susceptible DBM1Ac-S and field-evolved Bt-resistant DBM1Ac-R strains were provided by Drs. Jianzhou Zhao and Anthony (Tony) Shelton (Cornell University, USA) in 2003. Then, the near-isogenic Cry1Ac-resistant NIL-R strain was constructed in our laboratory in 2015 by six-time backcrossing between DBM1Ac-S and DBM1Ac-R along with Cry1Ac toxin selection. The lab-selected Cry1Ac-resistant SZ-R strain was collected at Shenzhen, China in 2003 and generated by continuous selection with Cry1Ac protoxin. The lab-selected Bt-resistant SH-R strain was collected at Shanghai in 2005 and was treated with a Bt var. kurstaki (Btk) formulation (WP with potency of 16000 IU/mg, provided by Bt Research and Development Centre, Agriculture Science Academy of Hubei Province, China). The DBM1Ac-R, NIL-R and SZ-R larvae present around 4500-, 5000-, and 500-fold resistance to Cry1Ac protoxin, while the SH-R strain presents approximately 2000-fold resistance to Btk formulation compared to the susceptible DBM1Ac-S strain.

Wild animals

The study did not involve wild animals.

Reporting on sex

The study was independent of sex.

Field-collected samples

The study did not involve samples collected from the field.

Ethics oversight

No ethical oversight was required as no vertebrate animals were involved in the study, and no ethical oversight of the experiments was required by our institution.

Note that full information on the approval of the study protocol must also be provided in the manuscript.
